# Defect engineered bioactive transition metals dichalcogenides quantum dots

**DOI:** 10.1038/s41467-018-07835-1

**Published:** 2019-01-03

**Authors:** Xianguang Ding, Fei Peng, Jun Zhou, Wenbin Gong, Garaj Slaven, Kian Ping Loh, Chwee Teck Lim, David Tai Leong

**Affiliations:** 1 Department of Chemical and Biomolecular Engineering, National University of Singapore, Singapore, 117585 Singapore; 2 Centre for Advanced 2D Materials, Graphene Research Centre, National University of Singapore, Singapore, 117546 Singapore; 3 Department of Physics, National University of Singapore, Singapore, 117542 Singapore; 4 Division of Advanced Nanomaterials, Suzhou Institute of Nano-Tech and Nano-Bionics, Chinese Academy of Sciences, Suzhou, 215123 China; 5 Department of Chemistry, National University of Singapore, Singapore, 117543 Singapore; 6 Department of Biomedical Engineering, National University of Singapore, Singapore, 117575 Singapore; 7 Mechanobiology Institute, Mechanobiology Institute, Singapore, 117411 Singapore; 8 Biomedical Institute for Global Health Research and Technology, Singapore, 117599 Singapore

## Abstract

Transition metal dichalcogenide (TMD) quantum dots (QDs) are fundamentally interesting because of the stronger quantum size effect with decreased lateral dimensions relative to their larger 2D nanosheet counterparts. However, the preparation of a wide range of TMD QDs is still a continual challenge. Here we demonstrate a bottom-up strategy utilizing TM oxides or chlorides and chalcogen precursors to synthesize a small library of TMD QDs (MoS_2_, WS_2_, RuS_2_, MoTe_2_, MoSe_2_, WSe_2_ and RuSe_2_). The reaction reaches equilibrium almost instantaneously (~10–20 s) with mild aqueous and room temperature conditions. Tunable defect engineering can be achieved within the same reactions by deviating the precursors’ reaction stoichiometries from their fixed molecular stoichiometries. Using MoS_2_ QDs for proof-of-concept biomedical applications, we show that increasing sulfur defects enhanced oxidative stress generation, through the photodynamic effect, in cancer cells. This facile strategy will motivate future design of TMDs nanomaterials utilizing defect engineering for biomedical applications.

## Abstract (web summary)

Transition metal dichalcogenide (TMD) quantum dots (QDs) have promising electronic properties which might be further tailorable by defect engineering. Here the authors describe a room temperature aqueous based synthesis of TMD QDs with controlled defect concentration, and demonstrate the correlation between defect concentration and biomedical activity.

## Introduction

Two-dimensional (2D) transition metal dichalcogenides (TMDs) with comparable structures to graphene, have attracted tremendous attention during the past few years. This large family of layered materials shared a common molecular formula and general structure where each member consists of mono-atomic thick stacked layers of repetitive covalently bonded X–M–X (M = Transition metal; X = Chalcogen). With decreasing number of layers, TMDs nanosheets transit from an indirect gap to a direct band-gap semi-conductor (e.g., 1.2–1.9 eV for MoS_2_ from bulk form to monolayer)^1,2^. This special layers-dependent physical property inspired 2D TMDs nanosheets-enabled applications in biomedicine^3,4^, sensors^5^, transistors^6^, catalysts^7,8^, photodetectors^9^, and energy storage devices^10,11^. Further reduction of the lateral size of TMDs few-layers or mono-layer nanosheets to quantum dots (QDs) or 0D nanodots further accentuate their electrical/optical properties due to a stronger quantum confinement and edge effects^12^. This enhancement introduced more dimensions of interesting and exploitable properties for catalytic and biomedical applications^13,14^, of which the latter is gravely under-represented. This could be due to the harsh and non-biocompatible methods in robust synthesis of many of the TMD QDs.

Atomic defects in transition metal dichalcogenides (TMDs) nanomaterials add an additional dimension to its optical, chemical and electronic activities. Top-down approaches like laser, plasma and electron bombardment have been intensively used to introduce defects on TMDs nanosheets and thereby controlling their optical and electrical properties. Moreover, the methods are usually single sheet process, albeit with great precision but at the expense of low scalability and high cost. Applying the same top–down approaches to reliably engineer defects in TMD QDs is extremely difficult due to the minute size of the QDs. This lack of defect engineering option has further curtailed TMD QDs’ potential.

In the past few years, many strategies, including lithium intercalation and exfoliation have been developed for synthesizing 2D TMD nanosheets^15–17^, but mild and general synthesis methods for 0D TMDs QDs are few. Top-down synthesis methods through a combination of grinding and sonication techniques, could obtain 1T-phase TMD QDs^11,12^. Although much progress has been achieved, to date, many more problems remain that needed solving before exploring their unique properties for catalytic and biomedical-based application. The top-down production of high quality TMD QDs has so far been extremely challenging because of the high degree of size and layers reduction required resulting in a low yield of production at the end of a long tedious process^18,19^, which involve time-consuming exfoliation and ultrasonication processes starting from bulk TMD crystals, exfoliated first to 2D TMD nanosheets and then further cutting them down to TMD QDs sizes. Fastidious post-treatment processes (for example, gradient centrifugation) is required; further reducing the yields of each sized TMDs QDs. Reports on bottom-up strategies for TMD QDs are generally limited to harsh hydrothermal methods (200 ^o^C and at least 24 h)^20–22^. Further defect engineering of as-synthesized TMD QDs will therefore allow for more property explorations of TMD QDs for catalytic, semi-conducting and biomedical applications.

Here we demonstrate a facile and possibly universal bottom-up route to synthesize MoS_2_ QDs with different degrees of defect under a fair degree of control. All the MoS_2_ QDs synthesized under our mild aqueous condition are of uniform sizes of around 3.9 nm. This synthesis method has been further expanded to successfully prepare a library of various other TMD QDs. We also showed, using MoS_2_ QDs, its potential as a photodynamic agent that is easily tunable through bottom-up disordering engineering. Previous studies have alluded to the possibility of defects in semiconductor QDs matter to photodynamic effect but that was never proven^23^, possibly due to the difficulty in controllable engineering defects in semiconductor QDs. In this paper, different degrees of defect engineering were achieved in 0D TMDs QDs via bottom-up stoichiometry deviations. With this defect-variable TMD QDs, the suspected correlation between defect degree and photodynamic properties is verified. The possible mechanism of defect enhanced reactive oxygen species (ROS) generation was proposed. By bottom-up defect engineering of TMD QDs, we showed that we could tune photodynamic oxidative stress effect on cancer cells.

## Results

### Characterization of as synthesized MoS_2_ QDs

The MoS_2_ QDs were prepared by a bottom-up route conducted in aqueous condition at room temperature via a simple chemical reaction starting with Na_2_S and MoCl_3_, MoCl_5_ or MoO_3_. Inspired by biomineralization, natural biopolymers such as BSA were used as surfactant because of their template effect and excellent biocompatibility^24,25^. Briefly, pH value of Mo-precursor solution was firstly adjusted to be above 11. In this high pH solution, the molybdenum precursors—MoCl_5_ or MoO_3_ decompose, yielding stable MoO_4_^2-^(Supplementary Figure 1). The reaction scheme can be expressed as:

$$4{\mathrm{MoCl}}_5 + {\mathrm{O}}_2 + 6{\mathrm{H}}_2{\mathrm{O}} + 8{\mathrm{OH}}^ - \to 4{\mathrm{MoO}}_4^{2 - } + 20{\mathrm{HCl}}$$

$${\mathrm{MoO}}_3 + 2{\mathrm{OH}}^ - \to {\mathrm{MoO}}_4^{2 - } + {\mathrm{H}}_2{\mathrm{O}}$$

**Fig. 1:**
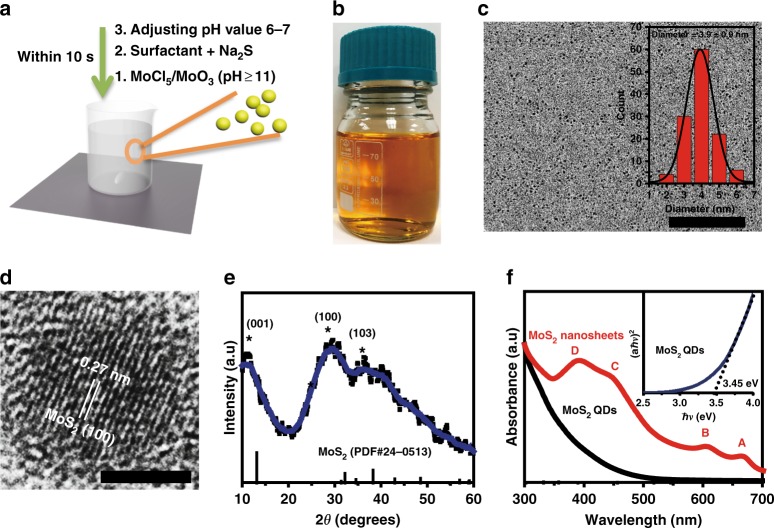
Benign aqueous room temperature bottom-up synthesis of MoS_2_ QDs. **a** Preparation of MoS_2_ QDs with bottom-up strategy. **b** Easy and scalable aqueous MoS_2_ colloidal suspension. Synthesis step takes less than 10–20 s (also refer to Supplementary Movie 1). **c** Ultrasmall but consistent as-synthesized MoS_2_ QDs. Inset: Distribution of MoS_2_ QDs size measured (ImageJ, *n* = 200). Scale bar: 100 nm. **d** HRTEM (Scale bar: 5 nm), **e** XRD and **f** UV-vis absorption spectra of MoS_2_ QDs showing a distinctively different nanomaterial from exfoliated MoS_2_ nanosheets

Then they were transferred into BSA solution; Na_2_S solution was introduced into the mixture. Subsequent adjusting of pH value to 6 with addition of HCl activates the sulfur precursors, initiating the reaction between MoO_4_^2−^ and sulfur precursors protected by BSA (Fig. 1a, Supplementary Movie 1). Our one-pot reaction was carried out under mild experimental conditions in the laboratory; without the need for high temperature and pressure or special apparatus^26^. Our method is easily scalable. The aqueous product showed a faint yellow color with good dispersibility (Fig. 1b). The transmission electron microscopy (TEM) images showed a consistent size quality of the as synthesized MoS_2_ QDs to be ~3.9 nm (Fig. 1c). The observed d-spacing (0.27 nm) is assigned to the (100) atomic plane of MoS_2_, confirming the high crystallinity of the MoS_2_ QDs (Fig. 1d). X-ray photoelectron spectroscopy (XPS) was also performed to measure the composition and the corresponding chemical valence of the elements in MoS_2_ QDs (Supplementary Figure 2). Typical Mo^4+^ 3d_3/2_ peaks of 232.8 eV and Mo^4+^ 3d_5/2_ peaks of 229.6 eV could be clearly observed from the XPS spectrum (Supplementary Figure 2a), which suggested the presence of Mo (IV) of MoS_2_ QDs^27^. Supplementary Figure 2b shows the binding energy of sulfur with the S 2p_3/2_ and S 2p_1/2_ peaks at 162.15 eV and 163.05 eV, which is consistent with −2 oxidation state of sulfur. The atomic ratio of Mo to sulfur was quantified to be 1: 2.31. The higher sulfur content compared to the initial feed ratio (Mo: S = 1:2) might be due to the presence of S- containing cysteines in BSA on the surface of MoS_2_ QDs. The structure of MoS_2_ QDs was further characterized by powder X-Ray diffractometry (XRD, Fig. 1e). The XRD pattern of MoS_2_ QDs shows broad diffraction peaks, which is in accordance with the XRD features of low-dimensional nanoparticles, suggesting the small grain sizes of the MoS_2_ QDs. The main peaks at 11^o^, 29^o^ and 36^o^ can be assigned to the characteristic (001), (100) and (103) planes of hexagonal 2H-MoS_2_ (JCPDS NO. 24-0513). The colloidal suspension of MoS_2_ QDs prepared was very stable, with no obvious aggregations over at least 3 months (Supplementary Figure 3). This high stability property is an important advantage in biomedical applications. In comparison to 2D MoS_2_ nanosheets with relatively large lateral dimensions, four distinct excitonic peaks from 2D MoS_2_ nanosheets (A, B, C and D) were absent in the optical spectrum of MoS_2_ QDs (Fig. 1f), along with strongly absorption blue-shifted towards shorter wavelengths. These optical features should result from the stronger quantum size effect of MoS_2_ QDs with decreased lateral dimensions relative to 2D MoS_2_ nanosheets (Supplementary Figure 4). Noticeably, MoS_2_ QDs exhibited a new absorption peak at around 310 nm. Similar absorption features were also observed by others^28,29^; attributed to their quantum confinement effect. Here the as-prepared MoS_2_ QDs had an average diameter of 3.9 nm, which is close to the bulk Bohr exciton diameter of ~4 nm^30^. The discretized bands induced by quantum confinement allowed transitions from the deep valance band to the conduction band, increasing the discrete absorption bands of MoS_2_ QDs. By using the method based on the relation of (*αћν*)^2^ versus *ћν* (*α* is absorbance, *ћ* is the Planck’s constant and *ν* is frequency), the optical band gap of our MoS_2_ QDs was calculated to be ~3.45 eV, which is higher than that of bulk and single-layer MoS_2_ (1.2–1.9 eV). The quantum confinement effect and surface states (like surface defects) induced by the bottom-up method in MoS_2_ QDs may have collectively contributed to this band gap. With the presence of localized defect states, the surface states can narrow the calculated optical band gap through generating band-tailing effects^31–33^.

### Surfactant effect on quality of MoS_2_ QDs

**Fig. 2:**
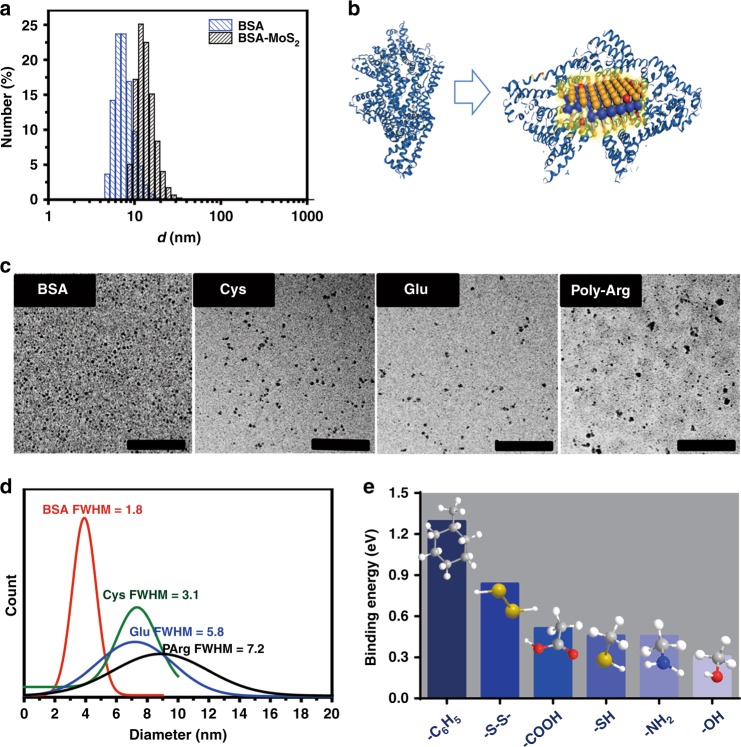
Surfactant effect on quality of MoS_2_ QDs. **a** Size of the BSA and MoS_2_ QDs determined by DLS. **b** Schematic illustration of possible BSA interactions with one MoS_2_ QD. **c** TEM images of MoS_2_ QDs synthesized with BSA, Cys, Glu and Poly-Arg. Scale bar: 200 nm. **d** Statistical analysis of the size distributions of MoS_2_ QDs synthesized with different biological surfactants. **e** Binding energy of major functional groups of BSA to MoS_2_ QDs

The hydrodynamic diameter of MoS_2_ QDs was determined to be 13 nm by dynamic light scattering (DLS), which was slightly larger than that of BSA (10 nm) and significantly higher than the TEM size of ~3.9 nm (Fig. 2a). One

MoS_2_ QD is likely to be interacting with one BSA molecule (Fig. 2b). The estimation value from thermogravimetric analysis was slightly larger than the DLS result (Supplementary Figure 5). With BSA as template to confine the growth of MoS_2_ QDs, the biomineralization process allow good size quality control. To investigate the role that the surfactant plays in the quality of MoS_2_ QDs, we also tested gluconate (Glu), poly-arginine (Poly-Arg) and cysteine (Cys) in the direct synthesis method. These biomolecules were selected as they provide the representative functional groups of BSA respectively: in Glu (–COOH), in Poly-Arg (–NH_2_), in Cys (–SH, –COOH, –NH_2_). Figure 2c suggests that all of above surfactants could mediate the successful synthesis of MoS_2_ QDs, while particle quality is highly dependent on the surfactant of choice. To quantify the particle size distribution, full width at half maximum (FWHM) was counted by analyzing the poly-distribution of MoS_2_ QDs from different surfactants. The size distribution and poly-distribution were analyzed from TEM samples with over 200 particles (with ImageJ). The statistical FWHM values of MoS_2_ QDs from BSA, Cys, Glu and Poly-Arg were 1.8, 3.1, 5.8 and 7.2, with the mean sizes of 3.9, 7.4, 7.3 and 9 nm, respectively (Fig. 2d), suggestive that BSA was more favorable for smaller QDs size and size distribution control. This also ruled out the sole fine size control of –COOH, –NH_2_ and –SH groups alone. Previous research suggested that disulfide bonds might possess much stronger binding energy to 2D MoS_2_ compared to single thiol^34,35^. To check that possibility in our MoS_2_ QDs, first-principle calculation was carried out to verify the binding affinity of the major functional groups of BSA to MoS_2_ QDs. As shown in Fig. 2e, benzene rings and disulfide exhibited much higher binding energy to MoS_2_ QDs compared to –SH, –COOH, –SH, –NH_2_ and –OH groups, which is 1.3 eV and 0.84 eV with respect to that of other groups (0.46 eV for –SH, 0.45 eV for –NH_2_ and 0.51 eV for –COOH) (Supplementary Table 1). These simulation results were consistent with the statistic FWHM values from TEM images (Fig. 2c), indicating that benzene rings and disulfide bonds may provide stronger affinity to stabilize QDs against aggregation and confine them for homogeneous growth, thus encouraging overall seeding and growth process in our case. To get direct experimental evidence that disulfide bonds does make a significant difference in the synthesis of MoS_2_ QDs, disulfide bridges of BSA was first reduced with NaBH_4_ to sulfhydryl groups. These denatured BSA (dBSA) with thiol groups were then used to prepare MoS_2_ QDs as per our synthesis protocol. The size of the as-acquired MoS_2_ QDs using dBSA exhibited a significant increase from 3.9 nm to a mean size of 6.3 nm (Supplementary Figure 6). This result was similar to Cys mediated samples which are laden with thiol groups, verifying the significant effect of disulfide bonds on the fine size distribution control of MoS_2_ QDs. The effect of benzene rings was also tested by using 2, 5 dihydroxybenzoic acid (DBA) as a stabilizer to synthesize MoS_2_-DBA. However, compared with other surfactants, the morphology of MoS_2_-DBA QDs was less controllable with the FWHM value to be 13.4 (Supplementary Figure 7), possibly due to the strong van der Waals interactions between benzene rings and weak ions-benzene ring interactions in the seeding stage of the particle formation. From these results we can conclude that the optimal surfactant for synthesizing MoS_2_ QDs are molecules containing disulfide bonds such as BSA.

The formation of MoS_2_ QDs was very fast at relatively low energy, as observed by TEM images sampled at different reaction time points and synthesis temperature (Supplementary Figure 8 and 9). The acquired MoS_2_ QDs could be easily purified by the addition of Cu^2+^ solution to precipitate out the MoS_2_ QDs, followed by centrifugation and dialysis to remove the supernatant and ions^36^ (Supplementary Figure 10), thus avoiding the fussy and time-consuming post-treatment process.

### Expansion of this synthetic strategy to other TMD QDs

**Fig. 3:**
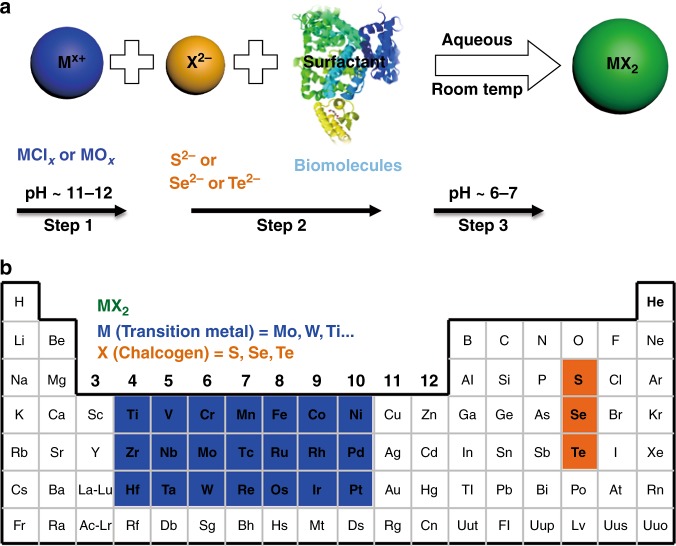
Illustration of the bottom-up synthesis of TMD QDs under mild condition. **a** In this study, only the synthesis of commonly reported TMDs (MoS_2_, WS_2_, RuS_2_, MoTe_2_, MoSe_2_, WSe_2_ and RuSe_2_) QDs were showed. **b** Extrapolating the synthesis method to other TMD QDs synthesis may present an universal method to synthesis the various TMDs QDs

**Fig. 4:**
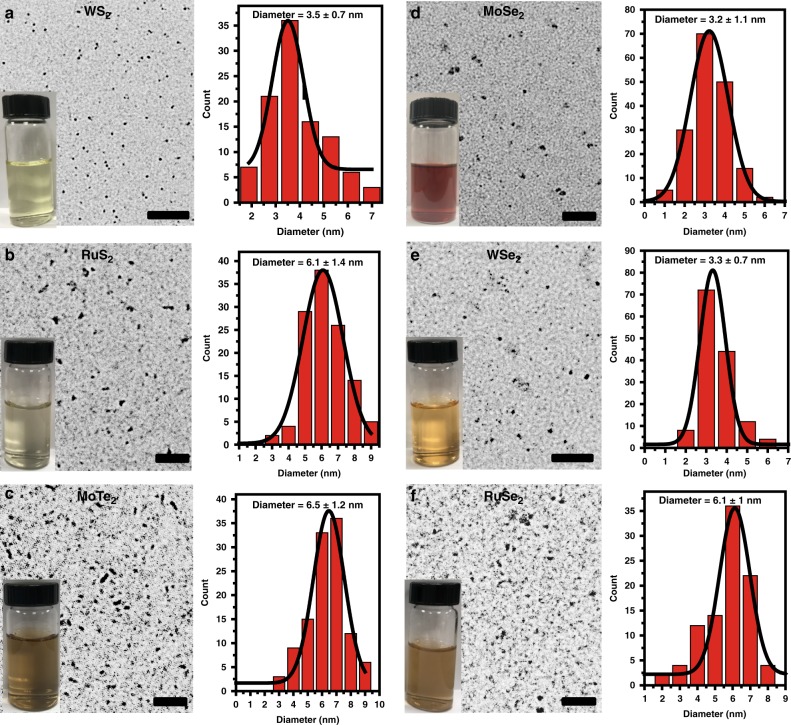
TEM images of other TMDs QDs synthesized under similarly mild conditions. **a**–**f** TEM images of WS_2_, RuS_2_, MoTe_2_, MoSe_2_, WSe_2_ and RuSe_2_ QDs. Insets: Stable colloidal suspension appearance of TMDs dispersed in aqueous condition. TMDs QDs have consistent sizes. Scale bar: 100 nm

We expanded this facile and mild bottom-up method to prepare a variety of TMD QDs based on the straightforward chemical

reaction between chalcogen precursor and transition metal salt such as metallic oxide or metallic chloride (Fig. 3a), which may be applied to synthesize various TMD QDs (Fig. 3b). To validate the feasibility of this method, BSA was applied as the surfactant for the proof-of-concept verification. Different transition metal sources (for example Mo, W and Ru) and chalcogenide source (S, Se and Te) were mixed with BSA at the pH value of 11. As suggested in Fig. 4, after pH value was adjust to 6 ~ 7. WS_2_, RuS_2_, MoTe_2_, MoSe_2_, WSe_2_ and RuSe_2_ were successfully prepared. The TEM clearly illustrated their highly homogeneous of size distribution with majority of the diameters to be below 10 nm. The corresponding images of different TMD QDs suspensions in H_2_O showed good dispersibility. Because of different bandgaps and sizes, the prepared TMD QDs library showed different colors in aqueous solution and exhibited correspondingly different UV-Vis

absorption spectra (Supplementary Figure 11). It is worth noting that some rod-like shape can also be found in the TEM images of MoTe_2_ samples, possibly due to high reactivity and instability of MoTe_2_ compounds compared to MoS_2_ /MoSe_2_ compounds^37^.

### Stoichiometry and characterization

**Fig. 5:**
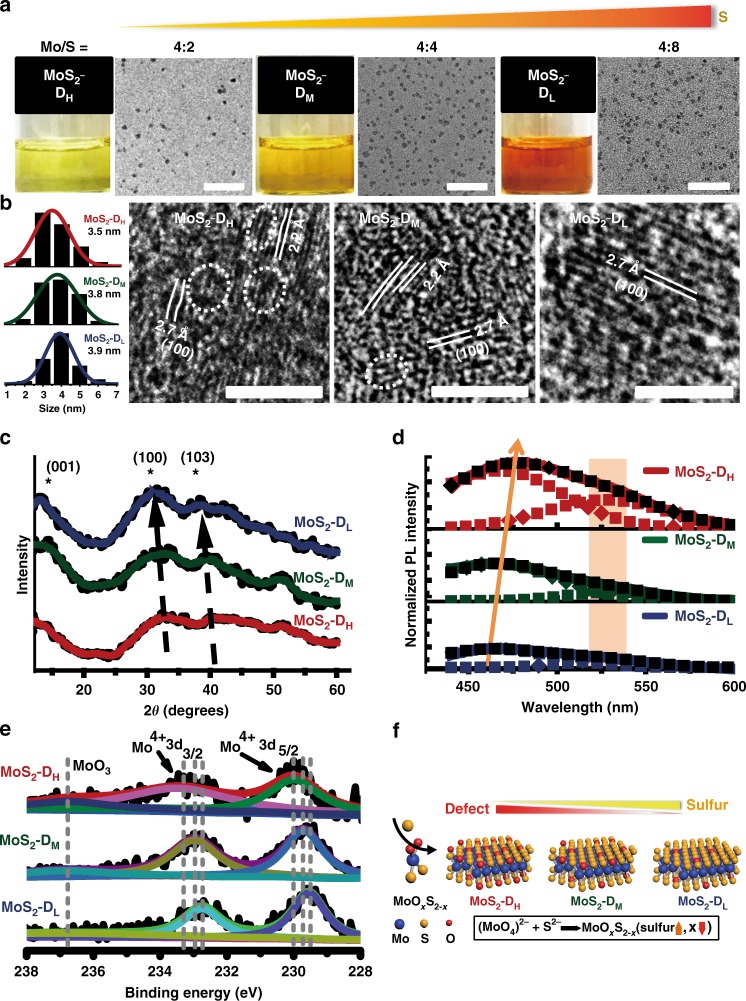
Engineering sulfur defects into MoS_2_ QDs through stoichiometric reaction control. **a** Adjusting precursor ratios produces three kinds of MoS_2_ suspension in pure water. TEM images show narrow size distribution. Scale bar: 50 nm. **b** Different stoichiometry does not affect overall size of QD MoS_2_. (Distributions derived from at least *n* = 200). HRTEM images show dislocations and distortions of lattice planes in MoS_2_ QDs due to intrinsic defects. Scale bar: 2 nm. **c** XRD show gradually enlarged lattice constants with decreasing stoichiometry in three MoS_2_ QDs. **d** PL spectrum show different emission intensities of the three MoS_2_ QDs at the same optical absorption. **e** XPS reveal the defect engineering was in the form of MoO_*x*_S_2-*x*_ by the substitution of oxygen for sulfur. **f** Structural model of the reaction pathway of MoS_2_ QDs and defect engineering in MoS_2_ QDs as a function of precursor stoichiometry

As our approach is bottom-up, the relative molarity of the reagents can be varied to be different from their stoichiometric ratios, enabling defect creation or dopant incorporation^38^. This theoretically allowed us to engineer defects in the as-synthesized TMD QDs from the bottom-up manner, thus providing a window of opportunity to tune their optical and electrical properties at the synthesis stage. Using MoS_2_ QDs as an example, three Mo/S relative molar concentrations, (4:2, 4:4 and 4:8) were pre-determined and reacted. Their respective products were named as MoS_2_-D_H_, MoS_2_-D_M_ and MoS_2_-D_L_ respectively. As shown in Fig. 5a, with increasing sulfur amount, the sample color changed from pale yellow for MoS_2_-D_H_ to yellow and orange for MoS_2_-D_M_ and MoS_2_-D_L_, respectively. Careful size analysis from TEM images (~200 particles counted) from the three groups indicated that varying initial relative concentration of reagents did not change the size distributions of the as-synthesized MoS_2_ QDs. Three MoS_2_ QDs groups exhibited similar size distributions with the average diameter of around 3.9 nm (Fig. 5b). The HRTEM images showed obvious discontinuities on the lattice planes such as dislocations and distortions were observed in the groups with the off-stoichiometric MoS_2_ S mole ratios like MoS_2_-D_H_ and MoS_2_-D_M_ QDs group (Fig. 5b). This discontinuity of the local lattice was thought to be caused by defects within crystals^39^. In MoS_2_-D_H_ HRTEM images, several lattice planes with more discontinuities presented in the MoS_2_ structures, suggesting the strongly disordered

arrangement of nanodomains in MoS_2_-D_H_ samples (Fig. 5b). When the sulfur amount increases approaching stoichiometric MoS_2_, relatively perfect single crystals without disorder were observed, suggesting the relatively fewer defects in MoS_2_-D_L_. (Fig. 5b) The XRD diffraction peaks in three samples were broadening, confirming the nanoscale of these crystallites in different dimensions (Fig. 5c). A slight shift of major diffraction peaks indexed to (100) and (103) toward lower angles could also be observed from MoS_2_-D_H_ to MoS_2_-D_L_ samples, indicating the gradually enlarged lattice constants with increasing sulfur amounts and the homogeneous phase structures of all three MoS_2_ QDs groups. The stoichiometry related surface states were also investigated by studying the PL behavior of three MoS_2_ QDs. All three defect states exhibited excitation-dependent PL behaviors (Supplementary Figure 12). Their representative PL spectrum, with each emission spectrum were normalized to their solution optical absorbance density at the PL excitation wavelength of 400 nm (Fig. 5d). Such normalization was used to highlight the PL intensity change. By integrating the respective peak areas, we observed that the quantum yield (QY) of MoS_2_-D_H_ (least sulfur content) possess the highest QY, which is approximately 1.79 times more than that of MoS_2_-D_M_ (medium sulfur content) and 2.42 times higher than that of MoS_2_-D_L_. Previous reports suggested that the surface state of nanomaterial is similar to a molecular state. Both the surface state (such as defects) and intrinsic state (quantum size effect) of nanomaterial contribute to the complexity of excited states of QDs^40,41^. The TEM analysis of the lattice planes disorder appears to point to MoS_2_-D_H_ as having the greatest degree of defects (Fig. 5). Interestingly, we observed a trend that with more defects, the photoluminescence quantum yield also increased. Moreover, the MoS_2_ QDs show two emission peaks with one at around 463–478 nm and the other one located at 530 nm, which could be attributed to intrinsic state emission (electron-hole recombination) and defect state emission, respectively^29^. In general, both intrinsic and defect state emissions influence the fluorescence spectrum. The red shift of fluorescence peaks from 463 nm of MoS_2_-D_L_ to 469 nm for MoS_2_-D_M_ and 478 nm for MoS_2_-D_H_ can be observed and possibly due to the gradually increasing defects from MoS_2_-D_L_ to MoS_2_-D_H_.

The controversy of whether defect sites and photoluminescence quantum yields are positively or negatively correlated is still ongoing. The influences of surface defects on photoluminescence quantum yields in smaller sized MoS_2_ quantum dots is still largely unknown. From our observations, the intrinsic state emission of the highest to lowest defect sites groups, MoS_2_-D_H_ to MoS_2_-D_L_ QDs still play the leading role in the PL emission and especially showed significantly higher efficiency in MoS_2_-D_H_ QDs. One of the possible explanation is the passivation effect from oxygen atoms in the MoS_2_ crystal lattice. In the presence of oxygen, the PL of CdSe QDs could be enhanced by as much as a factor of 6, resulting from the surface passivation by oxygen on nanocrystalline surfaces^42^. This phenomenon of oxygen atoms induced surface passivation of QDs has also been identified in many other semiconductor QDs^43,44^. The embedded oxygen atoms in the crystalline of MoS_2_ structures, presumably played two roles; first by creating sulfur distortion defects which support defect state emission, second by forming Mo–S–O bond on the crystalline surface, which passivate it thus enabling the intrinsic state emission enhancement. Preliminary experiment on the sulfur defect (in the form of Mo–O) reveal that the defect could increase PL intensity. However, substantial work such as ultrafast dynamics studies can to explore these photophysics of MoS_2_ QDs in greater detail.

To obtain more information about the intrinsic changes of surface states, high-resolution XPS can reveal the surface chemical environment of the MoS_2_ samples. Figure 5e shows two characteristic peaks located at ~ 229.6 and ~232.8 eV in three spectrums, which arose from the Mo^4+^ 3d_5/2_ and Mo^4+^ 3*d*_3/2_, suggesting the dominance of Mo (IV) in the MoS_2_ samples. Compared with MoS_2_-D_L_, samples with less sulfur amount show significantly broadened peaks from MoS_2_-D_M_ to MoS_2_-D_H_, confirming a higher degree of disorder in MoS_2_-D_H_^45^. In addition, both Mo^4+^ 3d_5/2_ and Mo^4+^ 3*d*_3/2_ doublet peaks in three samples shifted to lower binding energies with increasing sulfur amount, reflecting the reduction of MoS_2_. This behavior was consistent with decreasing intensity of Mo^6+^ peaks at ~236.2 eV, suggesting the presence of Mo–O bonds in MoS_2_-D_H_ decreased when sulfur amount approach to stoichiometric MoS_2_. These results indicate that the defect engineering was in the form of MoO_*x*_S_2-*x*_, by the substitution of oxygen for sulfur within the MoS_2_ lattices. This is also consistent with the XRD patterns that the substitution of the smaller O atom by the significantly larger S atom in the lattices induce the enlarged lattice constants (Fig. 5c). XPS quantification of the chemical compositions further revealed the atomic ratio of Mo to S varied from 1:1.8 to 1: 2.34 (Supplementary Figure 13). So as sulfur amount increases (MoS_2_-D_H_ group to MoS_2_-D_L_ group) resulted in the decreasing amount of oxygen atom in MoO_*x*_S_2_−_*x*_, giving the direct evidence of sulfur amount-dependent degree of defects in MoS_2_ QDs.

Collectively the combined studies above, the reaction pathway of MoS_2_ QDs can be revealed and the defective crystal structures have been proved to obtain via a MoO_4_^2−^ + S^2−^ → MoO_*x*_S_2−*x*_ pathway (Fig. 5f). The relatively lower sulfur amount was critically responsible for engineering the MoS_2_ QDs with S defects. With lower sulfur amount, the reaction became more insufficient, residual Mo−O bonds (originally from MoO_4_^2-^) that remained embedded within the otherwise crystalline MoS_2_ structure; thus creating distortion defects in the observed structure. This reaction behavior is similar to previous observation of controlling synthesis temperature in preparing defective 2D MoS_2_ nanosheets^39^. Here, 0D MoS_2_ QDs with controllable defect engineering were realized by simply tuning the synthesis reagent stoichiometry at room temperature.

### Surface vacancy associated singlet oxygen generation

**Fig. 6:**
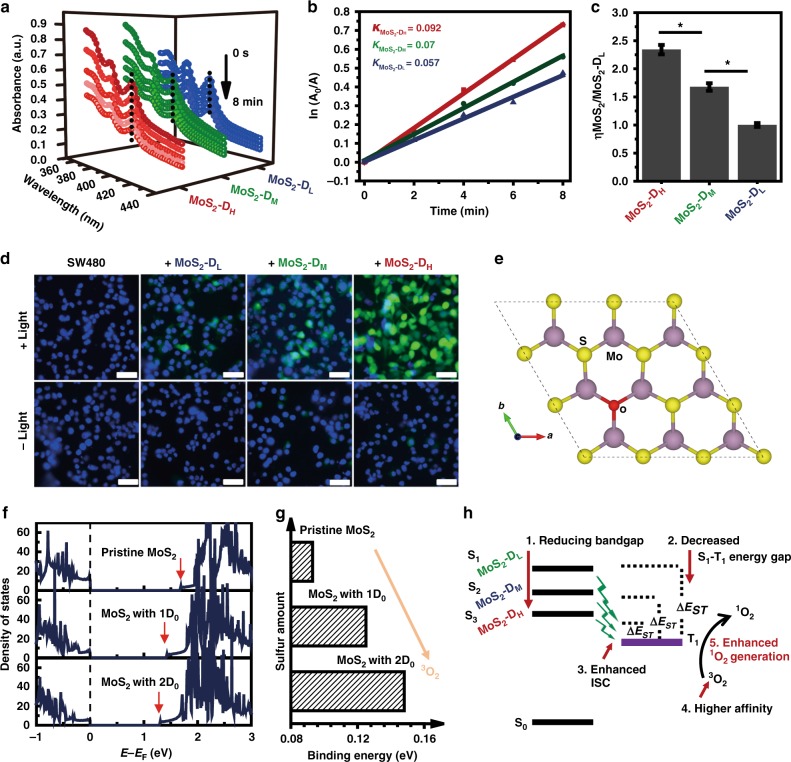
Positive correlation between sulfur defects and photodynamic efficiency in QDs. **a** Absorption spectra of three kinds of MoS_2_ QDs in the presence of ABDA under light irradiation (0.1 W cm-2, 8 min). **b** Typical decomposition rate of the photosensitizing process, where *A*_0_ is the absorbance of initial absorbance of ABDA and *A* is the absorbance of ABDA under light irradiation at different time points (Full data plots can be found as Supplementary Figure 19). **c** Relative ^1^O_2_ quantum yield of two MoS_2_ QDs groups relative to MoS_2_-D_L_ QDs. Mean ± SD, *n* = 4, Student’s *t*-test, p* < 0.05. **d** Detection of ROS generation at cellular level in colon cancer cell line, SW480 by intracellular ROS indicator H_2_DCFH-DA. Scale bar: 50 μm. **e** Structure illustration for the substitution of oxygen for sulfur within MoS_2_ lattices. **f** Calculated density of states of MoS_2_ QDs show the defects reduce the bandgap. **g** More sulfur defects induce stronger binding affinity of ^3^O_2_ to MoS_2_. **h** Proposed defect related ^1^O_2_ generation mechanism of MoS_2_ QDs

While the influence of defects on 2D TMDs have been largely studied for tuning optical and physical properties, which widely lead to excellent electronic applications, until now the defect effect on TMDs QDs are suggestive and speculative based on extrapolation from 2D TMDs observations and not applied directly in engineerable defects driven bioapplications. In addition, compared to 2D materials, 0D nanomaterials were supposed to exhibit better biological behavior than their 2D counterparts^46,47^. Thus photochemical related ^1^O_2_ generation capacity towards cancer therapy was investigated using our defect tunable MoS_2_ QDs. The singlet oxygen generation of the MoS_2_ was examined using 9, 10-anthracenediyl-bis(methylene) dimalonic acid (ABDA) as probe. It was found that without light irradiation, the absorbance of ABDA in three MoS_2_ QDs solution showed negligible decrease (Supplementary Figure 14). Under the white light irradiation, the absorption intensities within the groups decreased gradually with increased irradiation time (Fig. 6a). In addition, under the same irradiation condition without the presence of MoS_2_ QDs, both ^1^H-NMR and HPLC results did not show any appreciable change in the spectra of ABDA (Supplementary Figure 15), indicating the degradation of ABDA was indeed induced by the photosensitization from MoS_2_ QDs. The product of the ABDA trap after MoS_2_ QDs irradiation was checked by ^1^H- NMR and compared with the corresponding product of Rose Bengal (RB), a positive control known to generate ^1^O_2_ under irradiation. Similar chemical shifts were also observed in the corresponding H peaks of the products after irradiation (Supplementary Figure 16), suggesting that the species generated from MoS_2_ QDs and RB irradiation reacted with ABDA and produced similar ABDA products. We checked again with repeating the irradiation of MoS_2_-ABDA reaction in D_2_O or H_2_O conditions. It was found that there was higher depletion of the ABDA substrate when using D_2_O while irradiating MoS_2_ QDs (Supplementary Figure 17). This showed that ^1^O_2_ was generated from irradiation of MoS_2_ QDs. We further checked with a ^1^O_2_ 2, 2, 6, 6-tetramethylpiperidine (TEMP) sensor-electron spin resonance spectrum (ESR) assay. We found more product after 5 min of MoS_2_ irradiation (Supplementary Figure 18). Collectively, ^1^O_2_ was likely generated after MoS_2_ irradiation.

To compare the ^1^O_2_ quantum yield of three MoS2 QDs upon light irradiation, calculation was performed based on the photochemical methods reported before^48–50^. The decreased OD at 400 nm (OD_400_) was performed to determine the decomposition rate constants of the photosensitizing process of three MoS_2_ QDs, which was obtained by fitting the various OD_400_ curves (Fig. 6b). By further integrating the optical absorption of MoS_2_ QDs in the range 400–800 nm, the ^1^O_2_ quantum yield of two kinds of MoS_2_ QDs relative to MoS_2_-D_L_ were determined. The MoS_2_-D_H_ and MoS_2_-D_M_ groups respectively exhibited ^1^O_2_ quantum yield of approximately 2.3 times and 1.7 times of MoS_2_-D_L_ QDs’ quantum yield (Fig. 6c, Supplementary Figure 19). Physical quenching between ^1^O_2_ and N’s lone pair electrons of amines may exist; especially those aromatic amines of the BSA-surfactant^51^. It is therefore important to check that the differences in ^1^O_2_ quantum yield is not due to different amounts of BSA that are on the surface of the three kinds of MoS_2_ QDs. We quantified the amount of BSA on the surface of the three kinds of MoS_2_ using the micro-BCA protein assay. The BCA protein based assay showed no significantly different amounts of BSA on the same amount of the three MoS_2_ QDs defect types (Supplementary Figure 20). This indicated that the physical quenching due to proteins on the surface of the three MoS_2_ QDs groups are similar. Thus, confirming that the significant increase in the ^1^O_2_ quantum yields of MoS_2_-D_M_ and MoS_2_-D_H_ over MoS_2_-D_L_ is due to increasing defects.

To further prove the correlations between the degree of defects and photodynamic efficiency, the ROS generation capacity was further detected inside the cancer cells by using dye dichlorohydrofluorescein diacetate (DCFH-DA) as an intracellular ROS indicator. DCFH-DA is non-fluorescent but can be oxidized by ROS to yield highly fluorescent-dichlorofluorescein (DCF). As shown in Fig. 6d, after irradiation, strongest green fluorescence was observed in MoS_2_-D_H_ incubated groups, with relatively lower green fluorescence in MoS_2_-D_M_ groups and the lowest green fluorescence in MoS_2_-D_L_, which clearly confirmed the positive correlation between the degree of defects of MoS_2_ QDs and ROS generation capacity.

QDs acting as photosensitizers used in photodynamic therapy have attracted great attention in the area of nanomedicine^52–57^. In a typical photosensitizing process, the photosensitizer absorbs a quantum of light from ground state (S_0_) to the excited singlet state (S_1_), which is further converted to the excited triplet state (T_1_) via intersystem crossing (ISC). The subsequent energy transferring from T_1_ to triplet oxygen (^3^O_2_) result in the formation of singlet oxygen (^1^O_2_)^58^. The quantum yield of ^1^O_2_ generated by photosensitizers can be expressed as equation (1)^59^

1 $$\phi _\Delta = \phi _{\mathrm T}\phi_ {\rm{en}},$$

where *Ф*_T_ is the quantum yield of T_1_ formation and *ϕ*_en_ is the efficiency of energy transfer from T_1_ to ^3^O_2_. To understand the enhanced ROS generation mechanism in cells, we analyzed the relationship between the defects and corresponding ROS generation behavior in MoS_2_ QDs. Previous records have confirmed that *Ф*_T_ is determinate by the ISC rate constants *k*_ISC_, which could be estimated from Eq. (2)^60^

2 $$k_{{\rm{ISC}}} \propto \frac{{({\mathrm T}_1|H_{\rm SO}|{\mathrm S}_1)^2}}{{\Delta E_{\rm S1 - T1}}}$$

Here *H*_SO_ represents the Hamiltonian for the spin-orbit perturbations and $\Delta E_{\rm S1 - T1}$($\Delta E_{\rm ST}$) is the energy gap between S_1_ and T_1_. Therefore by engineering the HOMO-LUMO energy level in molecules to reduce the energy gap Δ$E_{\rm ST}$, high photosensitizing efficiency could be achieved^61,62^. In our MoS_2_ QDs experiments, we speculated that the sulfur vacancy derived defects engineered the bandgap of MoS_2_ QDs, thus modulating the $\Delta E_{\rm ST}$ to affect the photosensitizing capacity. To clarify this hypothesis, theoretical simulation using DFT was performed to check the density of states (DOS) of MoS_2_ QDs. After constructing a 3 × 3 × 1 supercell structure, zero, one and two sulfur atoms in the supercell were removed and substituted by oxygen atoms (Fig. 6e). This leads to different degrees of sulfur defects in MoS_2,_ which denoted as pristine MoS_2_, MoS_2_-1*D*_0_ and MoS_2_-2*D*_0._ The calculated DOS in Fig. 6f demonstrated that the vacancies of sulfur atoms and the substitution of oxygen atoms act as n-type dopants. The more substitution of more electronegative oxygen atoms led to more charge carriers, with narrower bandgap from pristine MoS_2_ of 1.68 eV to 1.42 eV (MoS_2_-1*D*_0_) and 1.30 eV (MoS_2_−2*D*_0_). This decreasing bandgap of the simulated defective mode in MoS_2_ QDs qualitatively agrees with experimental observations from optical band gap (Supplementary Figure 21). Our calculation indicate that the sulfur defects in MoS_2_ QDs tend to reduce the bandgap with lowering Δ$E_{\rm ST}$, which improved ISC efficiency and account for the observed enhanced ^1^O_2_ generation in the photosensitizing process.

On the other hand, the substitution of sulfur atom with the more electronegative oxygen atom modulated the charge density distributions in MoS_2_ crystals, affecting the Gibbs free energy for ^3^O_2_ adsorption. To understand the binding affinity after different degrees of defect, the binding energies of ^3^O_2_ on three MoS_2_ QDs were calculated by DFT. As shown in Fig. 6g, the binding energies for oxygen adsorption on MoS_2_ QDs decrease with less sulfur defect. The binding energies of ^3^O_2_ on MoS_2_-2*D*_0_ are 0.15 eV, which are much larger than MoS_2_-1*D*_0_ (0.13 eV) and pristine MoS_2_ (0.09 eV). Moreover, with three sulfur atoms substituted by oxygen atoms, binding energy further increased to 0.17 eV. This result suggested that a higher defect MoS_2_ QD possessed a stronger binding affinity toward ^3^O_2_ adsorption, which may allow higher oxygen coverage on the surface of MoS_2_ QD in the energy transfer process, thus paving the way for higher *ϕ*_en_ in ^1^O_2_ generation.

Besides the reduced bandgap and the strengthened binding affinity between MoS_2_ and ^3^O_2_ (Fig. 6h), the spin-orbit perturbations (*H*_SO_) in defective MoS_2_ QDs could also affect the intersystem crossing in the photosensitizing process. The vibronic coupling involved in Mo–S and Mo–O bonds would be significantly increased due to the increasing degree of defects. The decrease in size increases edge sites and dangling bonds in MoS_2_ QDs. This further enhances the likelihood of vibrational modes hence promoting the intersystem crossing. The real exciton splitting situation due to the presence of defects and quantum confinement effect of typical semiconductor nanocrystals behavior in MoS_2_ QDs’ case complicated their energy state. Here the proposed singlet and triplet states of MoS_2_ QD structure models was under the consideration of surface molecular states of MoS_2_ QDs. Further investigations to understand the electronic structure and exciton of MoS_2_ QDs are warranted.

### Defect-dependent photodynamic therapy to kill cancer cells

**Fig. 7:**
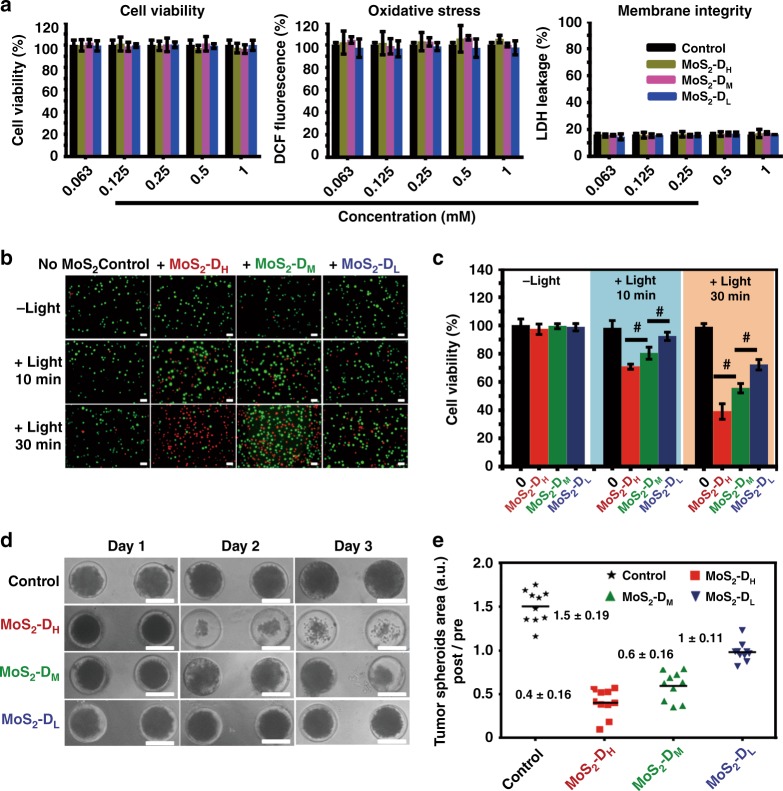
MoS_2_ QDs with more S defects produce more oxidative stress in cancer cells. **a** MoS_2_ QDs treatment (without light) on human endothelial cells (HMVEC) showed negligible cytotoxicity, negligible excessive ROS generation and low apoptosis induction. The measurements were performed in triplicate. **b** Calcein AM and PI co-staining and **c** cell viabilities of SW480 cells incubated with different defect laden MoS_2_ QDs under white light exposure. The measurements were performed in triplicate. **d** MoS_2_-D_H_ showed the highest size reduction of 3D tumor spheroids assay. This implied high penetration of QDs into the interior of the 3D tumor spheroids mass with the highest defect group producing the highest oxidative stress that killed the cells. **e** Quantification of 3D tumor spheroids area post treatment and laser excitation with MoS_2_-D_H_ showing the greatest cell death amongst the three MoS_2_ groups. Mean ± SD, *n* = 10, Student’s *t* test, *p*^#^ < 0.05. Scale bar: 100 μm

Inspired by defect-dependent ROS generation capacity, the photodynamic efficiency of the three MoS_2_ QDs for killing cancer cells were further conducted. The material biocompatibility was first investigated. Cell viability, LDH and ROS level were examined to systematically evaluate the cytotoxicity effect (biocompatibility) of three MoS_2_ QDs using endothelial cells as a model for a non-cancer cell type which would be in contact with introduced MoS_2_ QDs bioapplications. Fig. 7a suggest that no obvious toxicity of all the three MoS_2_ QDs on human microvascular endothelial cell (HMVEC) cells at MoS_2_ QDs concentration as high as 1 mM for 24 h, suggesting good biocompatibility of all three MoS_2_ QDs types. There was no additional upregulation of ROS as the MoS_2_ QDs was not irradiated with light. When increasing the dose of MoS_2_ QDs, there was no increasing LDH leakage due to loss of membrane integrity as a result of induced apoptosis. We next used MoS_2_ QDs as photosensitizers in photodynamic cancer cell therapy in an in vitro model. Using SW480 cells as model cancer cells, Calcein AM (green fluorescence) and propidium iodide (PI, red fluorescence) staining were performed to visualize live/dead cells in the cancer cell therapy process. Control cell group with light treatments only or MoS_2_ QDs incubation only (without light treatment) maintained high cell viability (Fig. 7b). As expected, cells incubated with higher defects MoS_2_-D_H_ QDs showed considerably lower cell viability under light irradiation compared to MoS_2_-D_M_ and MoS_2_-D_L_ treatment groups (Fig. 7c). After being irradiated for a short 10 min, only 72% of cells incubated with MoS_2_-D_H_ were viable, which further decreased to 39.7% when the irradiation time prolonged to 30 min. At the same treatment concentrations, cell viability of MoS_2_-D_M_ and MoS_2_-D_L_ groups decreased from 81% to 55.2 and 92% to 72% respectively, as the irradiation time was prolonged from 10 min to 30 min.

These results demonstrated that MoS_2_ QDs with higher defects are more effective photodynamic agents to kill cancer cells over those with lower defects at the same concentration.

To further test the photodynamic efficiency in vitro on cancer cells, 3D SW480 colorectal tumor spheroids were used as a more realistic cancer model to simulate the therapeutic responses of three MoS_2_ QDs groups^63,64^. To examine the photodynamic effect, 3D tumor spheroids were incubated with different MoS_2_ QDs groups for 6 h to allow for increased penetration and then irradiated with white light for 30 min. After three days, as expected, the size of tumor spheroids incubated with MoS_2_ QDs with a single irradiation show higher degree of growth retardation compared to control group without MoS_2_ treatment (Fig. 7d, e, Supplementary Figure 22). More importantly, the MoS_2_-D_H_ treated groups show the greatest tumor spheroids size reduction, as compared with that of the MoS_2_-D_M_ and MoS_2_-D_L_ groups at equivalent MoS_2_ concentrations. These results indicated that the degree of defects was related to the photodynamic efficiency of MoS_2_ QDs, showing the possible defect engineering in QDs to enhance their therapy effect while reducing their actual dosage used in clinic, thereby preventing the potential chronic side effects and complications.

## Discussion

In summary, we have demonstrated the biomineralization assisted bottom-up strategy for synthesizing a wide range of QDs from the TMD family with using mild conditions. The strategy employed straightforward chemical reactions between sodium chalcogenides and transition metal chlorides or transition metal oxides with a high yield reaction within a matter of tens of seconds. Disulfide bonds were shown to be the functional group for size quality control, and further verified by DFT simulations. The major benefit of the synthetic route is the ability of controllable defects engineering on TMD QDs via tuning precursor stoichiometry. Our studies revealed that the reaction pathway of MoS_2_ QDs and the defective crystal structures might be $({\mathrm{MoO}}_4)^{2 - } + {\mathrm{S}}^{2 - } \to {\mathrm{MoO}}_{{x}}{\mathrm{S}}_{2 - {{x}}}$. This synthetic strategy simplifies the synthetic process with mild conditions, enriching the TMD QDs library for exploring their physical and chemical properties and related applications. Our bottom-up strategy for synthesizing TMDs QDs provided an ideal platform for investigating the correlation between the surface defects of semiconductor nanomaterials and the corresponding capacity of anti-cancer oxidative stress generation. Strongly positive correlation between degrees of sulfur defects and photodynamic efficiencies could be observed in the prepared MoS_2_ QDs. The density of states and molecular dynamics calculations suggest that the sulfur defect in MoS_2_ QDs reduced the bandgap and strengthened the binding affinity between MoS_2_ QDs and ^3^O_2._ This may have contributed to the intersystem crossing and energy transfer separately in the photosensitizing process; highlighting the significant potential for defect engineering as an intrinsic alteration tool used in conjunction with existing TMDs bionanotechnologies^65–67^ without adding yet another material.

## Methods

### Materials

All chemicals and reagents were used as received without any further purification. MoCl_5_ (95%), MoO_3_ (95%) and Bovine Serum Album (BSA) were purchased from Sigma-Aldrich Co. (USA). Deionized water was used throughout the synthesis.

### Sample characterization

The morphologies and size of MoS_2_ QDs samples were characterized by TEM (FE-TEM; JEOL JEM-2100F, Japan). Nanoparticle sizes were determined by measuring no <200 randomly selected nanoparticles from TEM micrographs with ImageJ (http: //rsbweb.nib.gov/ij/). The powder XRD measurements were performed using a Bruker D8 advanced diffractometer with a Cu Ka irradiation in the 2*θ* range of 20^0^-60^0^. The elemental composition and binding energy of the sample were characterized by X-ray photoelectron spectroscopy (XPS; AXIS HIS, Kratos Analytical). The absorbance spectrum scanning and fluorescence intensities were conducted using microplate reader (BioTEK H4FM, USA). Fluorescence imaging were taken with inverted fluorescence microscope (Leica DMI6000, Germany) and the phase contrast images were captured by inverted microscope (Olympus-CX41, Japan).

### Synthesis of MoS_2_ quantum dots

In a typical procedure, Mo-precursor solution was firstly prepared by dissolving MoCl_5_ or MoO_3_ into dH_2_O by adjusting the pH value to be 11. The resultant solution was transparent and colorless after sonication. Then 1 mL of Mo precursor solution mixed into 39 mL of BSA solution (1 mg/mL) followed by adding 0.2 mL 0.5 M Na_2_S solution under vigorous stirring at room temperature. The pH value of the mixed solution was subsequently adjusted to 6~7 by adding 1 M HCl. After neutralizing the pH, a clear yellow solution of MoS_2_ quantum dots was produced quickly.

### Synthesis of other TMD quantum dots with BSA

The protocol of synthesizing other TMD quantum dots was similar to MoS_2_ quantum dots. The only difference was the preparation of Se- and Te- precursors. In a typical preparation of Se precursor, 79.9 mg of Se powder was added into 1 mL of NaBH_4_ solution (80 mg) to reduce it at ambient condition. 30 min later, black Se powder disappeared completely and the clear solution was Se precursor. For the preparation of Te precursor, 31.9 mg of Te powder was added into 200 μL NaBH_4_ solution (28.4 mg). After 30 min, black Te powder fully disappeared and the clear solution was Te precursor. For the synthesis of WS_2_, RuS_2_, MoTe_2_, WSe_2_ and RuSe_2_, similarly mild conditions were carried as MoS_2_ above.

### Photodynamic evaluation of three MoS_2_ QDs with ABDA

In the experiments, ABDA as the probe of ^1^O_2_ was added to different MoS_2_ QDs dispersion solution with final concentration of 10 μM. Then the mixture solution was transferred to a cuvette and exposed to white light (400–800 nm, 100 mW) perpendicularly for 8 min. The light source irradiated a region of 1 cm^2^. For the convenience of observation, the OD of three MoS_2_ QDs at 400 nm was adjusted to the same. The absorbance change at 400 nm was recorded at various time points to obtain the degradation rate of ABDA. For the comparison of ^1^O_2_ quantum yield, all the ^1^O_2_ quantum yield of MoS_2_ was normalized to that of MoS_2_-D_L_, relative ^1^O_2_ ƞ to MoS_2_-D_L_ using the following equation:

$$\frac{{{\mathrm{MoS}}_2}}{{{\mathrm{MoS}}_2 -{\mathrm{D}}_{\mathrm{L}}}} = \frac{{K_{{\rm{MoS}}_2}A_{{\rm{MoS}_2 - D_L}}}}{{K_{{\rm{MoS}_2 - D_L}}A_{{\rm{MoS}}_2}}},$$

where $K_{{\rm{MoS}}_2}$ and $K_{{\rm{MoS}_2 - D_L}}$ represent the decomposition rate constants of the photodegradation of ABDA with different MoS_2_, which were determined by plotting Log_e_(Abs_0_/Abs) versus irradiation, where Abs_0_ is the initial absorbance of ABDA, Abs is the ABDA absorbance at different irradiation time. $A_{{\rm{MoS}}_2}$ and $A_{{\rm{MoS}_2 - D_L}}$ refer to the light absorbed by different MoS_2_ and MoS_2_-D_L_ respectively, which are determined by integration of the optical absorption bands in the wavelength range from 400 to 800 nm.

### Intracellular ROS assay

DCF-DA probed the ROS generation inside cells. SW480 cells were cultured on 8-well cell chamber slides overnight. Overnight medium was replaced with fresh medium containing different MoS_2_ QDs (at 0.5 mM). After incubation for 6 h, DCF-DA (25 μM) was added for 15 min, and then either left in the dark or irradiation with white light (100 mW cm^−2^) for 5 min. After washing and staining the cells with Hoechst 33342 (5 μg/mL), fluorescence images of the cells were captured using Nikon A1 confocal microscope (Nikon, Japan).

### Photodynamic killing of SW480 cancer cells

To assess the photodynamic effect of MoS_2_ QDs on SW480 cells, SW480 cells was seeded and cultured in 96-well plates overnight. Then different groups of MoS_2_ QDs were used to treat SW480 cells for 6 h. Subsequently, cells were irradiated with white light (100 mW cm^−2^) for 10 and 30 min. After treatments, the cells were further cultured for 12 h. Cell viability after different treatments was evaluated by WST assays and Tali Image based Cytometer (Life Technologies, USA).

### Formation and photodynamic efficiency in SW480 colorectal tumor spheroids

To examine the photodynamic effect on 3D SW480 colorectal tumor spheroids, 3D cell spheroids were first prepared. The solidified agrose micro-molds were sterilized by UV irradiation for 30 min and then equilibrated with DMEM medium for 12 h. After that 50 μL of SW480 cell suspension were seeded into each agarose micro-mold, 5 min after the cells settle down into the micro-mold, 500 μL of DMEM medium was added to the well. After 24 h, 3D SW480 cell spheroids were formed due to gravity and the aggregation of cells. For photodynamic therapy treatment, the different MoS_2_ QDs were added into 3D SW480 cell spheroids. After 6 h, 3D SW480 cell spheroids were either exposed to dark or white light (100 mW cm^−2^) for 30 min. The images of 3D SW480 cell spheroids were captured by a light microscope (Olympus-CX41, Japan) in the subsequent days and the size of the 3D SW480 cell spheroids were measured with ImageJ software.

### Density functional theory (DFT) binding energy siumlation

Adsorptions of various chemical groups on MoS_2_ were investigated by performing simulations at the density functional theory (DFT) level as implemented in the Vienna ab initio simulation package (VASP)^68^, with the exchange-correlation functional of Perdew-Burke-Ernzerhof (PBE)^69^. The long-range van der Waals interactions were calculated within the Tkatchenko and Scheffler scheme to avoid the empirical parameters^70^, while the self-consistent screening and polarizability contraction effects were also taken into account, in view of their important roles in determining the weak inter-molecular interactions^71^. The MoS_2_ layer was modeled with a vacuum region of 20 Å. The first Brillouin zone was sampled with a *k*-point mesh with a plane-wave cutoff of 450 eV. The various molecules including CH_3_–X (X = –OH, –SH, –NH_2_, –COOH, –SSCH_3_ and –C_6_H_5_) and CH_3_–CONH–CH_3_ were simulated following the equation $E_{{\rm{ad}}} = E_{{\rm{MoS}}_2 + {\rm{group}}} - E_{{\rm{group}}} - E_{{\rm{MoS}}_2}$ with the results summarized in Supplementary Table 1.

### Methodology for density of states (DOS) of models MoS_2_

- DFT based VASP.
- PAW-PBE potential electron-ion interaction and exchange-correlation.
- 3 × 3 × 1 supercell structure is applied
- 500 eV for plane-wave expansion cutoff.
- 7 × 7 × 1 Gama-centred k-point mesh.
- All structures were relaxed until the force is smaller than 0.001 eV/Angstrom with a total energy convergence criterion of 1 × 10^−6^ eV.
- Vacuum is 15 Å.
- The relaxed lattice parameter for MoS_2_ monolayer is 3.190 Å

## Supplementary information

Supplementary Information

Peer Review File

Description of Additional Supplementary Files

Supplementary Movie 1

## Data Availability

All the relevant data are available from the corresponding authors upon reasonable request.
