# Supplementary material for: Defect engineered bioactive transition metals dichalcogenides quantum dots
Source: Nat Commun. 2019 Jan 3;10:41. doi: 10.1038/s41467-018-07835-1 (PMC6318297; doi:10.1038/s41467-018-07835-1)
Supplement: Supplementary file 1 — Supplementary Information [file 41467_2018_7835_MOESM1_ESM.docx]

**SUPPLEMENTAL INFORMATION**

**Defect Engineered Bioactive Transition Metals Dichalcogenides Quantum Dots**

**Defect Engineered Bioactive Transition Metals Dichalcogenides Quantum Dots**

*Xianguang Ding^1,2^, Fei Peng^1^, Zhou Jun^3^, Wenbin Gong^5^, Garaj Slaven^2,3^, Kian Ping Loh^2,6^, Chwee Teck Lim^2,4,7,8^* and David Tai Leong^1^**

^1^Department of Chemical and Biomolecular Engineering, National University of Singapore, Singapore 117585, Singapore

^2^Centre for Advanced 2D Materials, Graphene Research Centre, National University of Singapore, Singapore 117546, Singapore

^3^Department of Physics, National University of Singapore, Singapore 117542, Singapore

^4^Department of Biomedical Engineering, National University of Singapore, Singapore 117575, Singapore

^5^Division of Advanced Nanomaterials, Suzhou Institute of Nano-Tech and Nano-Bionics, Chinese Academy of Sciences, Suzhou 215123, China

^6^Department of Chemistry, National University of Singapore, Singapore 117543, Singapore

^7^Mechanobiology Institute, Mechanobiology Institute, Singapore 117411, Singapore

^8^Biomedical Institute for Global Health Research and Technology, Singapore 117599, Singapore

*Correspondence: [cheltwd@nus.edu.sg](mailto:cheltwd@nus.edu.sg) (D.T.L.)

*Correspondence: [ctlim@nus.edu.sg](mailto:ctlim@nus.edu.sg) (C.T.L.)


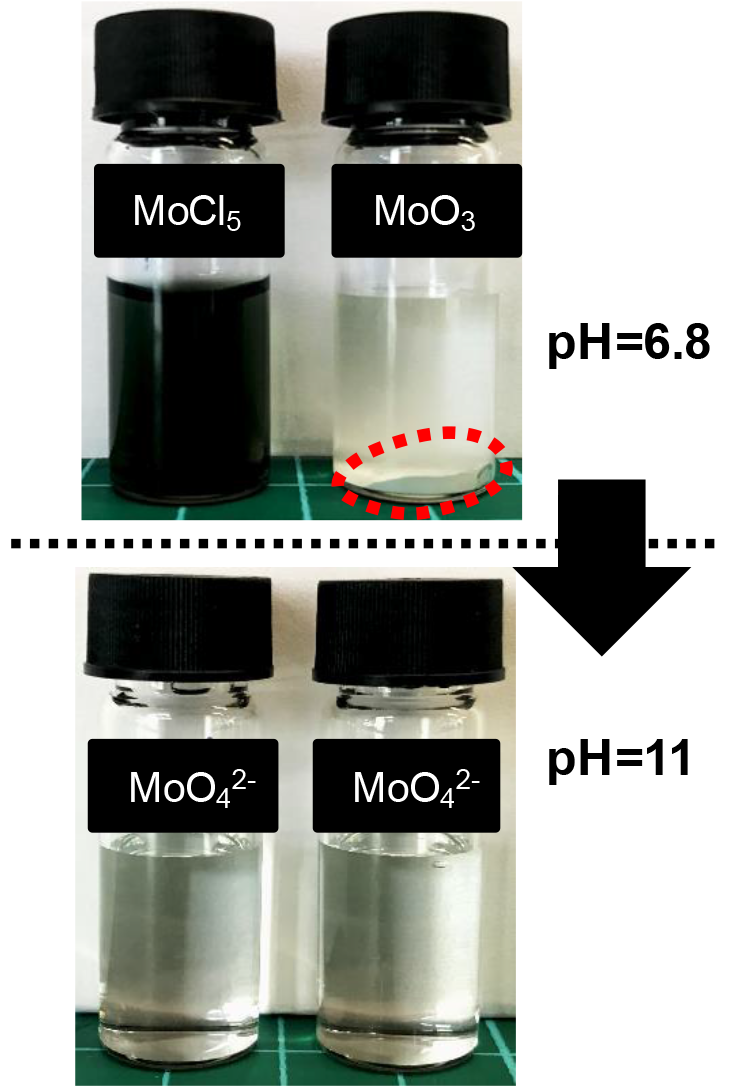


Supplementary Figure 1. MoCl_5_ and MoO_3_ dissolve to a colorless stable MoO_4_^2-^ solution at high pH. Red circle demarcates insoluble MoO_3_ at pH 6.8.


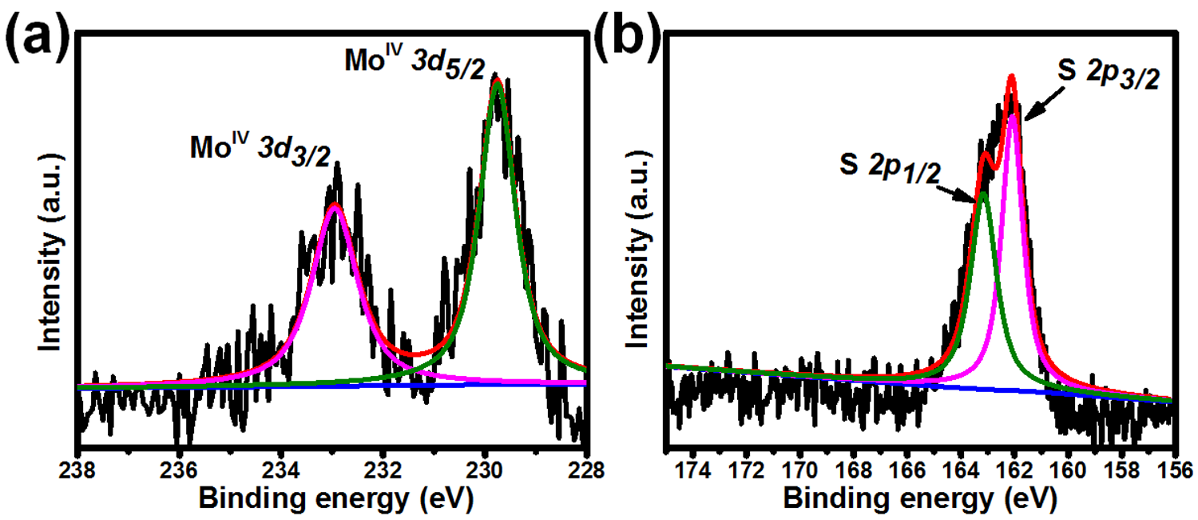


Supplementary Figure 2. XPS spectra of as-prepared MoS_2_ QDs: (a) Mo and (b) S.


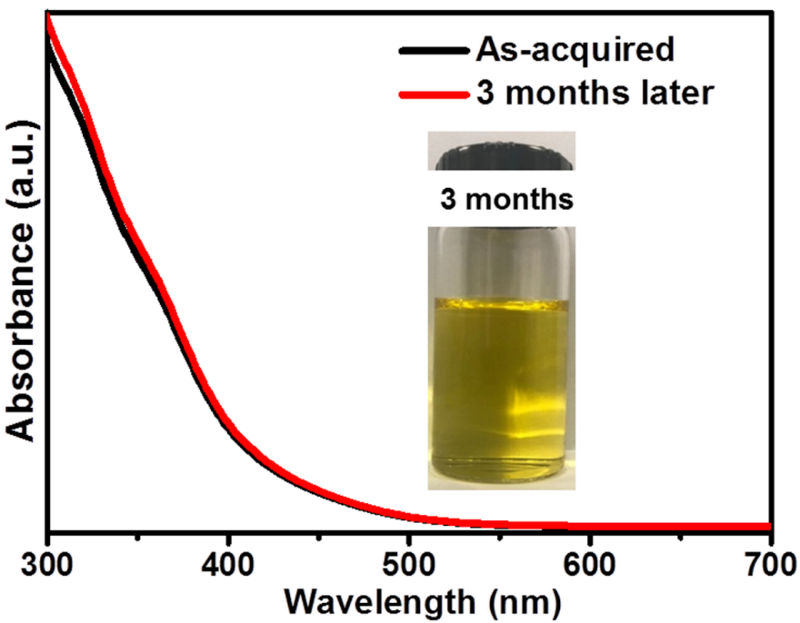


Supplementary Figure 3. High storage stability of aqueous dispersion of MoS_2_ QDs.


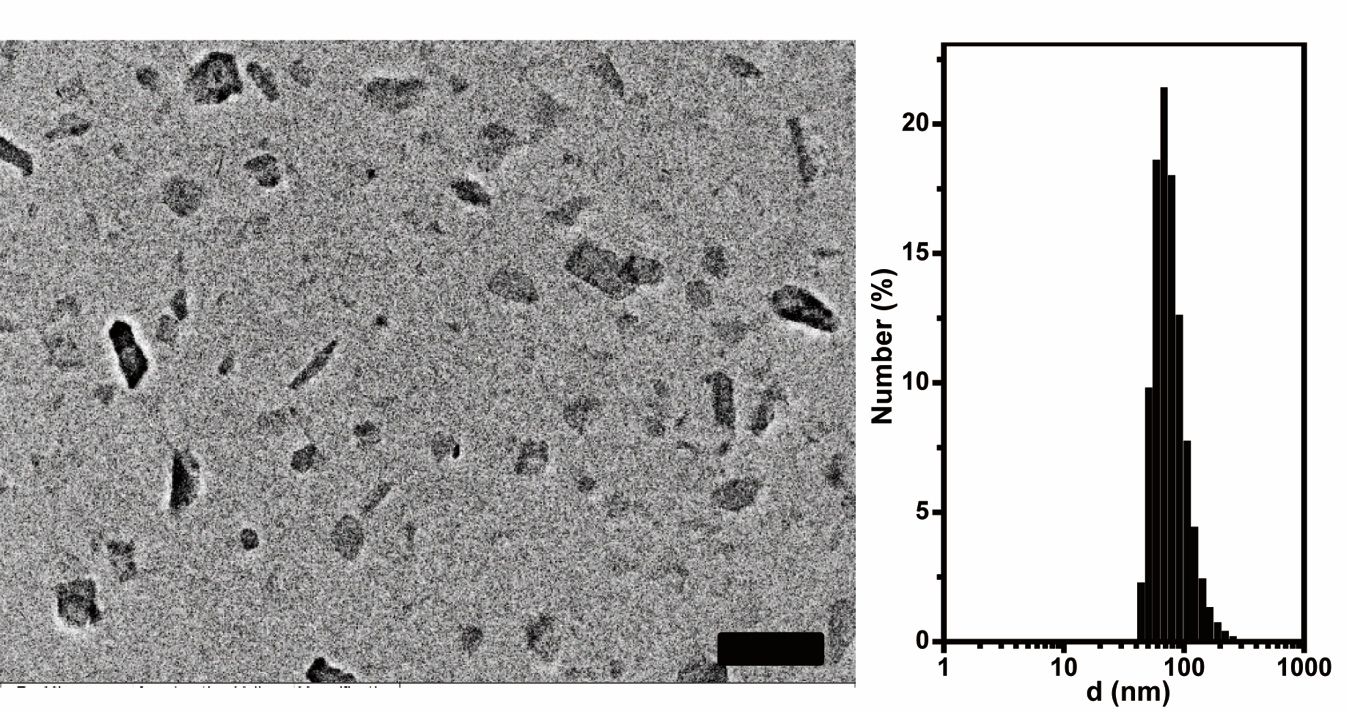


Supplementary Figure 4. TEM image of MoS_2_ sheet exfoliated by using BSA. Scale bar: 100 nm.

*
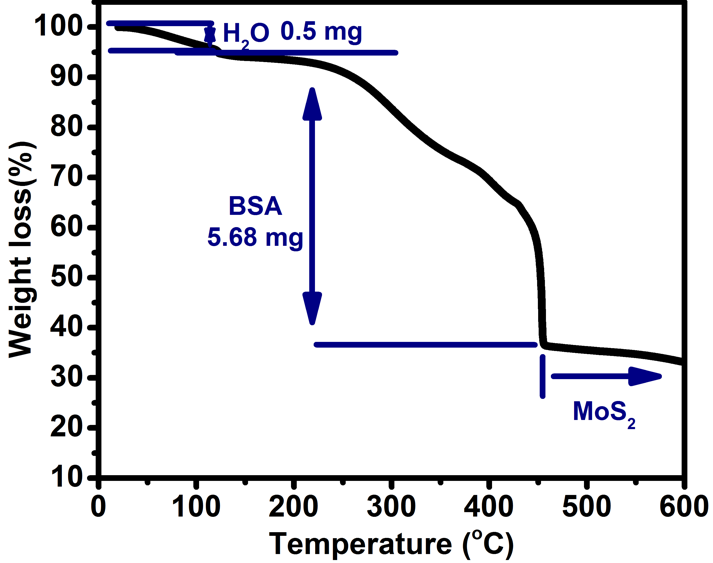
*

**Supplementary Figure 5.** TGA of BSA-coated MoS_2_ QDs performed under inert nitrogen atmosphere.

Analyzing the result from TGA curves, three stages was observed: weight loss below 100 ^o^C can be assigned to water; the weight loss from 200 ^o^C to 450 ^o^C is mainly due to the thermal decomposition of decomposed of BSA. The weight loss from 450 ^o^C is correspond to the thermal decomposition of MoS_2_ (*Composites Part A: Applied Science and Manufacturing* 94 (2017): 1-9.). The incompletely decomposed carbonaceous product under N^2^ atmosphere at 450 ^o^C was about 20 % of the total BSA amount according to the previous BSA report (*Journal of colloid and interface science* 389.1 (2013): 31-41). Then the total amount of BSA and MoS_2_ was calculated to be around 7.1 mg and 2.4 mg, separately.

The mass of per 3.9 nm MoS_2_ QDs (based on TEM results) and BSA molecule were further calculated:

$m_{\mathrm{Mo}S_{2}}$= $\frac{4}{3}\pi r^{3}$ x $\rho_{MoS_{2}}$ = $\frac{4}{3}\pi{(1.95 nm)}^{3}$ x 5.06 g/cm^3^ = 1.5 x 10^-19^ g MoS_2_/QD

$m_{\mathrm{BSA}}$ = $\frac{{MW}_{BSA}}{6.022 x {10}^{23}}$ = 1.1 x 10^-19^ g BSA/Molecule

Then the ratio of $N_{BSA}$ to $N_{MoS_{2}}$ was calculated as:

$\frac{N_{\mathrm{BSA}}}{N_{\mathrm{Mo}S_{2}}}$ = $\frac{{7.1}/{m_{\mathrm{BSA}}}}{{7.4}/{m_{\mathrm{Mo}S_{2}}}}$ = 4.03

The ratio estimated from TGA result (4.03) was slightly greater than the DLS measurements but still at the same order of magnitude. Considering that the simple calculation above is based on a single size QDs average diameter, the actual QDs size distribution may also affect the number of BSA ligand binding on the surface.

| *Group* | **-OH** | **-NH_2_** | **-COOH** | **-C_6_H_5_** | **-S-S-** | **-SH** |
| --- | --- | --- | --- | --- | --- | --- |
| $E_{{MoS}_{2}+group}$ **(Ha)** | -21730.435 | -21730.572 | -21843.732 | -21886.118 | -22490.650 | -22053.312 |
| $E_{Group}$ **(Ha)** | -115.614 | -95.746 | -228.903 | -271.260 | -875.809 | -438.485 |
| $E_{{MoS}_{2}}$ **(Ha)** | -21614.81 | -21614.810 | -21614.810 | -21614.810 | -21614.810 | -21614.810 |
| $E_{ad}$ **(Ha)** | -0.011 | -0.017 | -0.019 | -0.047 | -0.031 | -0.017 |
| $E_{ad}$ **(eV)** | -0.304 | -0.453 | -0.510 | -1.29 | -0.835 | -0.455 |

**Supplementary Table 1.** Calculated binding affinities of the major functional groups in BSA to MoS_2_ QDs.

$$E_{ad}= E_{{MoS}_{2}+group}- E_{group}- E_{{MoS}_{2}}$$

where $E_{group}$,$E_{{MoS}_{2}}$ and $E_{{MoS}_{2}+group}$are the energies of the bound functional group, the MoS_2_ surface and the complex of MoS_2_ and functional group respectively.

The oxygen adsorption energies on the pristine and oxygen-atom doped MoS_2_ were also calculated. The oxygen-doped MoS_2_ was modeled by substituting oxygen atom for sulfur. The nearest three sulfur atoms around the oxygen adsorption site were replaced one by one to describe the doping level. All the structures were optimized by using the conjugate gradient method, in which the convergence for total energy and interaction force was set to be 10^-5^ eV and 2×10^-3^ eV/Å, respectively.


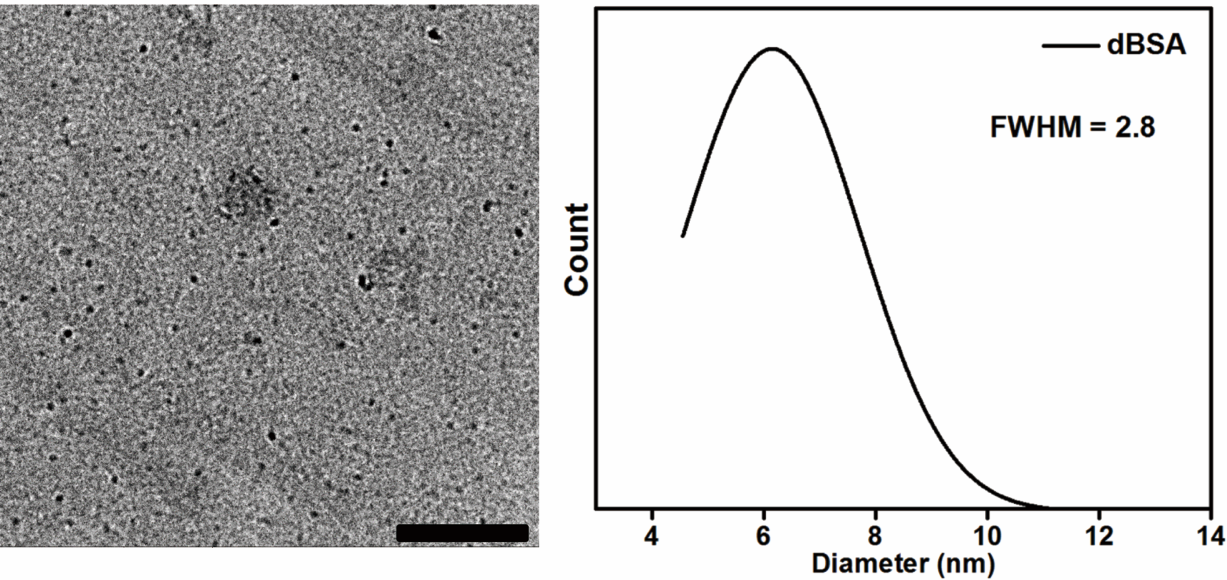


**Supplementary Figure 6.** TEM images and size distribution of MoS_2_ QDs synthesized with dBSA. Scale bar: 100 nm.


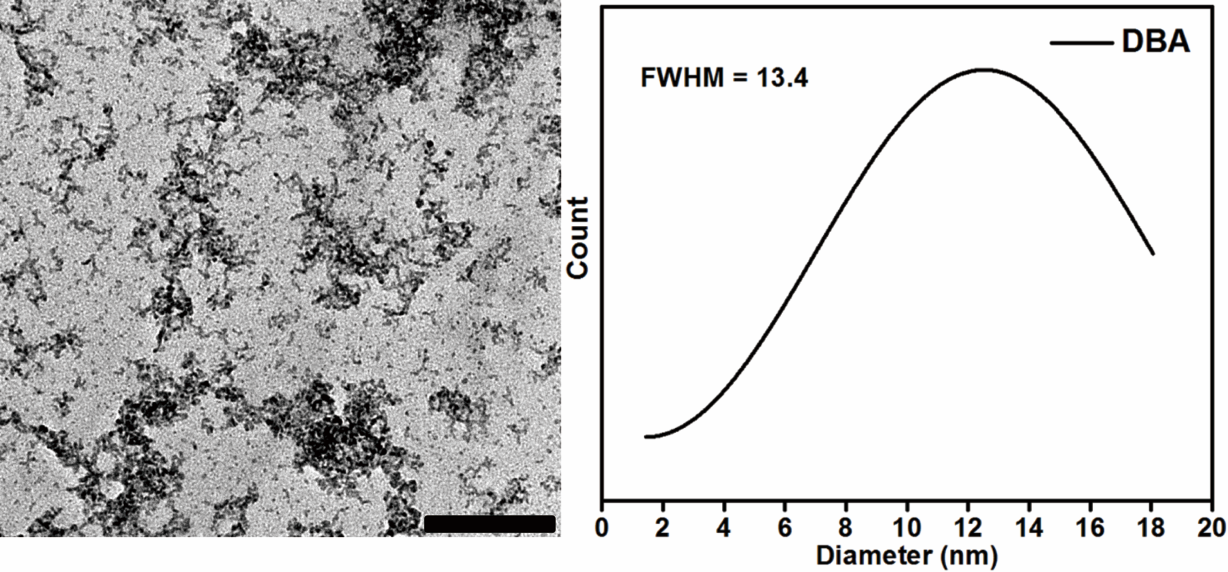


**Supplementary Figure 7.** TEM images and size distribution of MoS_2_ QDs synthesized with DBA. Scale bar: 100 nm.


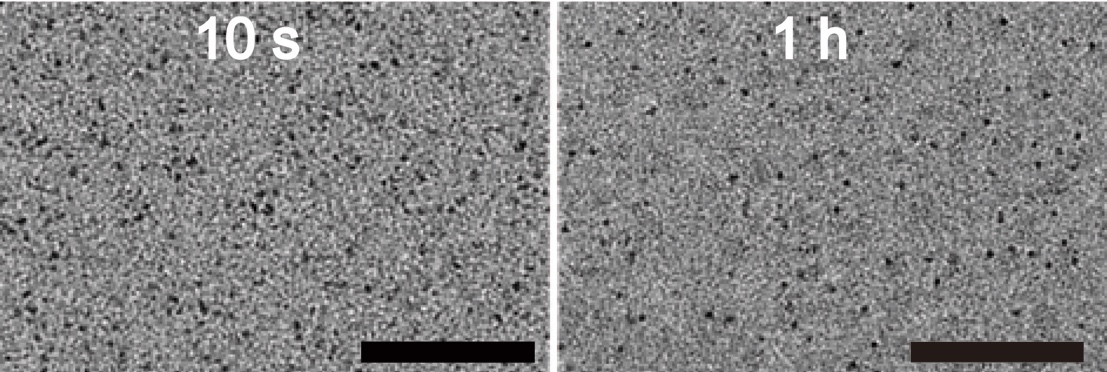


**Supplementary Figure 8.** TEM images of MoS_2_ QDs sampled at the reaction of 10 s and 1 h showing in-discernible differences even with extended reaction time. Scale bar: 100 nm.


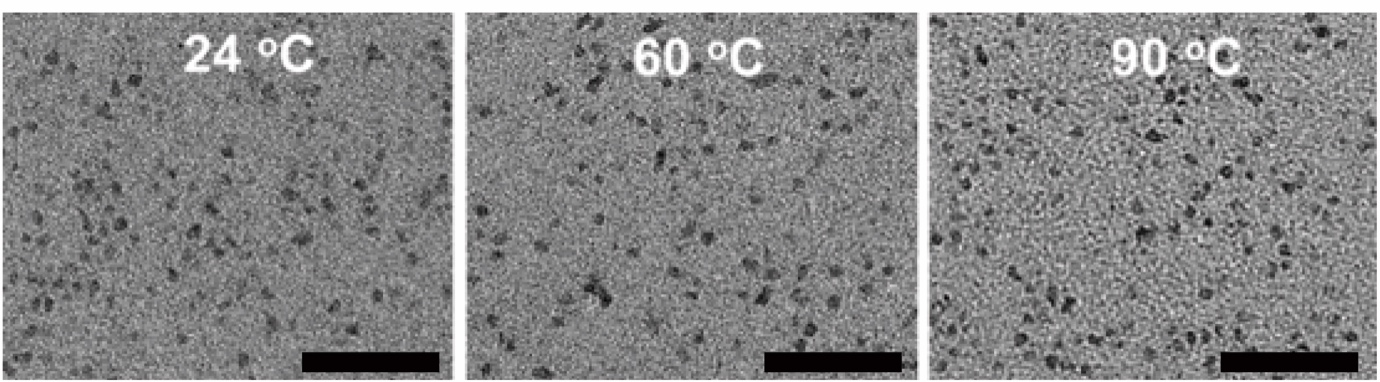


**Supplementary Figure 9.** TEM images of MoS_2_ QDs synthesized at the 24 ^o^C, 60 ^o^C and 90 ^o^C showing in-discernible differences even with elevated reaction temperature. Scale bar: 50 nm.


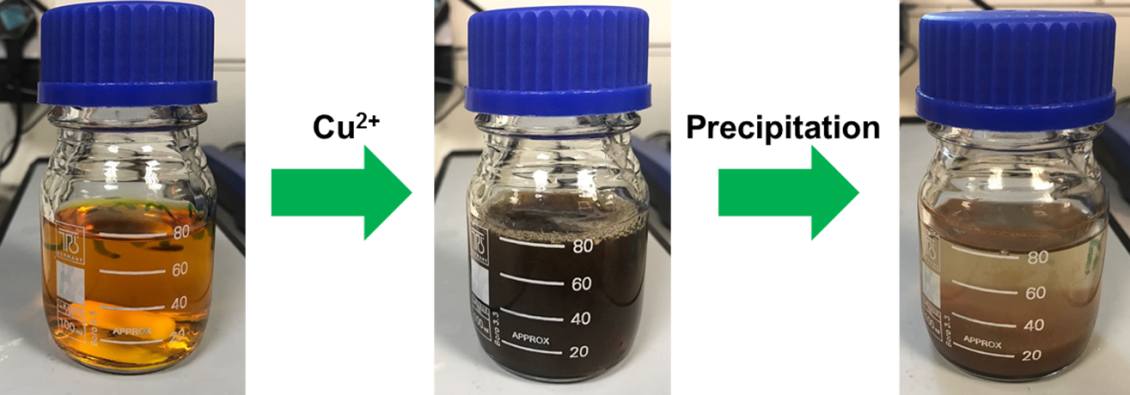


**Supplementary Figure 10.** Schematic illustration of the purification process of MoS_2_ QDs by adding Cu^2+^.


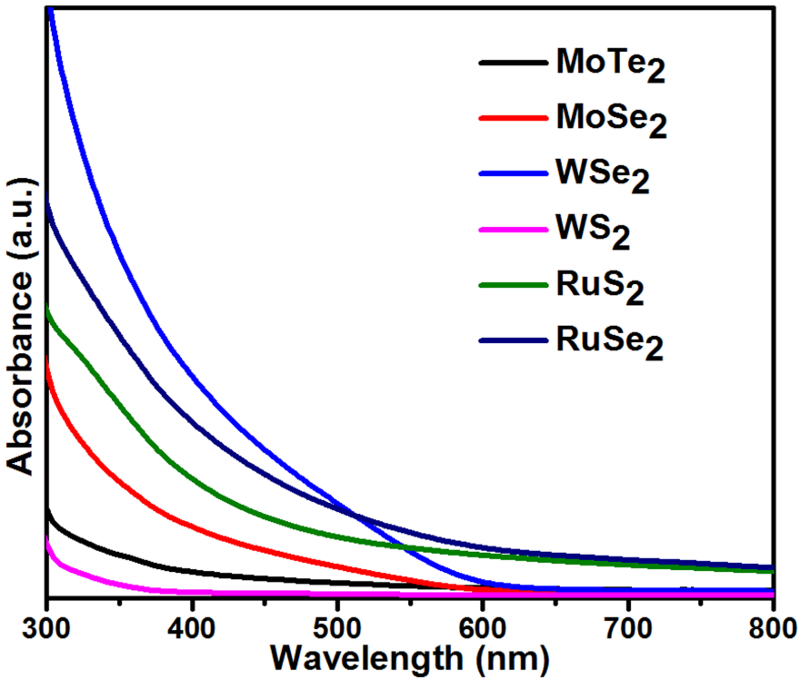


**Supplementary Figure 11.** UV-vis spectra of MoTe_2_, MoSe_2_, WSe_2_, WS_2_, RuS_2_ and RuSe_2_ in aqueous condition.


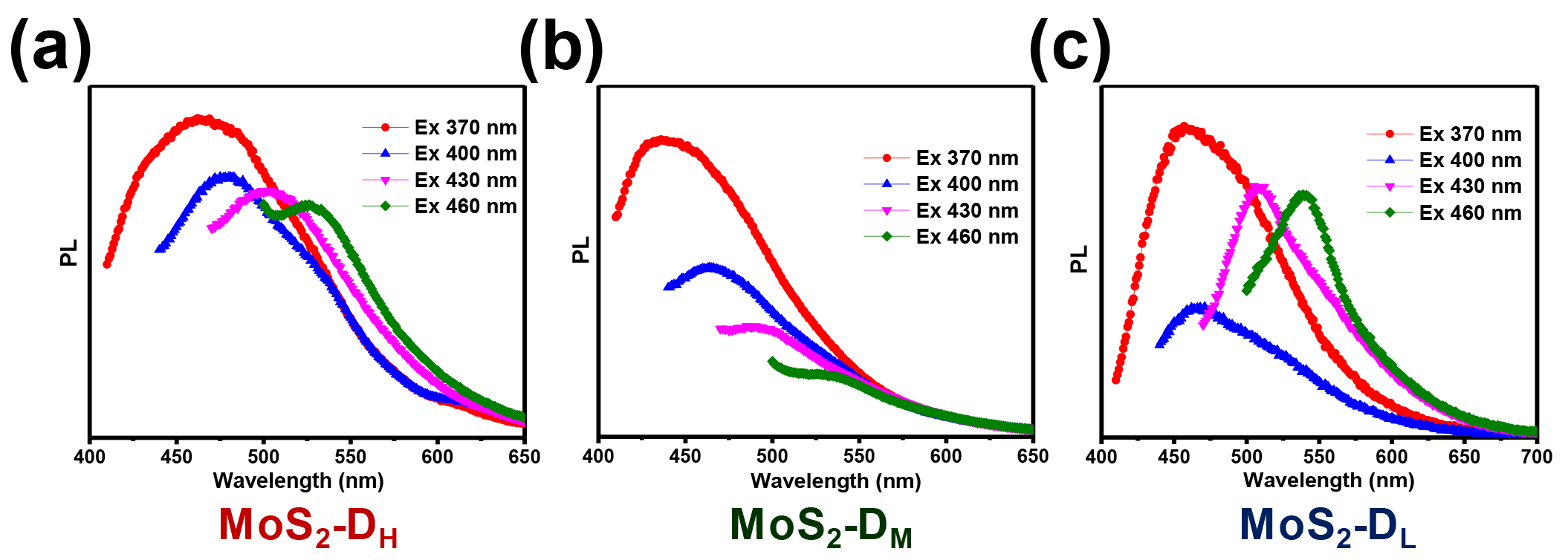


**Supplementary Figure 12.** The excitation-dependent PL behaviors of the MoS_2_-D_H_, MoS_2_-D_M_ and MoS_2_-D_L_ QDs.

|  | **Atomic ratio of Mo/S by XPS** |
| --- | --- |
| **MoS_2_-D_H_** | **1: 1.8** |
| **MoS_2_-D_M_** | **1: 2.29** |
| **MoS_2_-D_L_** | **1: 2.34** |

**Supplementary Figure 13.** Elemental analyses of the MoS_2_-D_H_, MoS_2_-D_M_ and MoS_2_-D_L_ QDs.


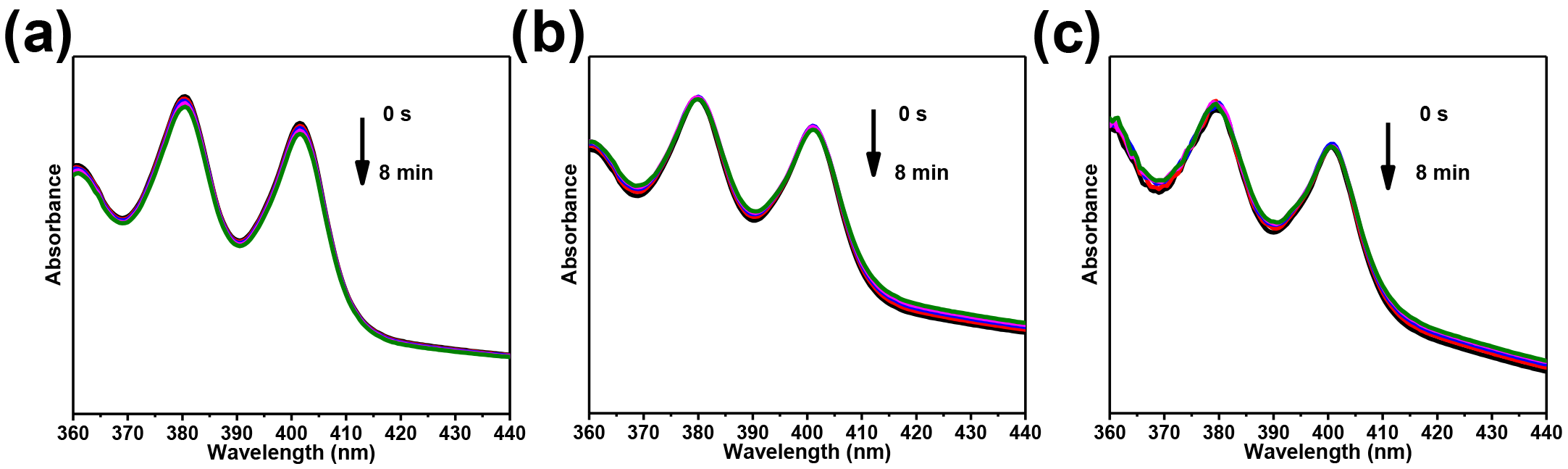


**Supplementary Figure 14.** Absorption spectra of three kinds of MoS_2_ QDs in the presence of ABDA without light irradiation: (a) MoS_2_-D_H_, (b) MoS_2_-D_M_ and (c) MoS_2_-D_L_


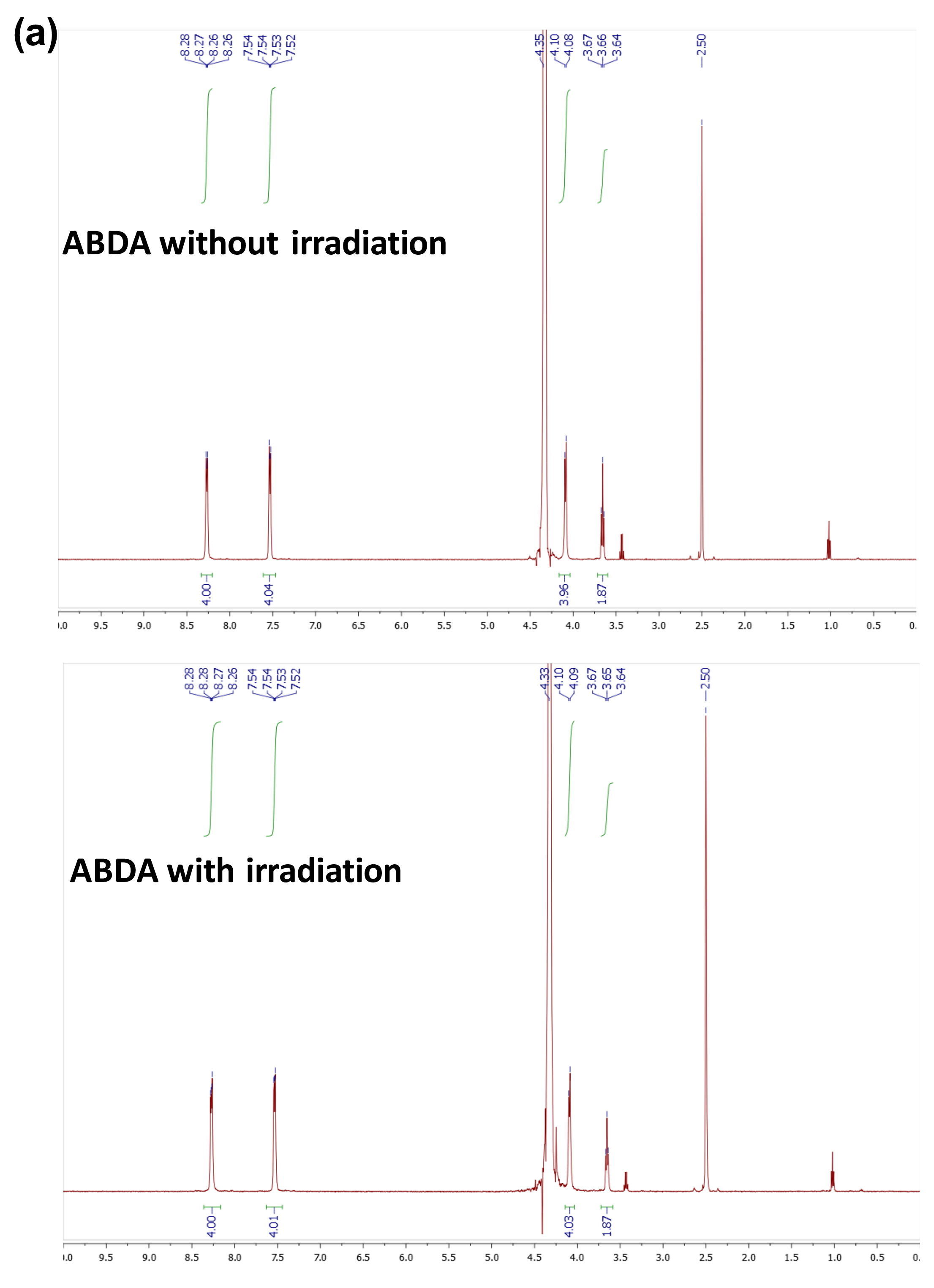


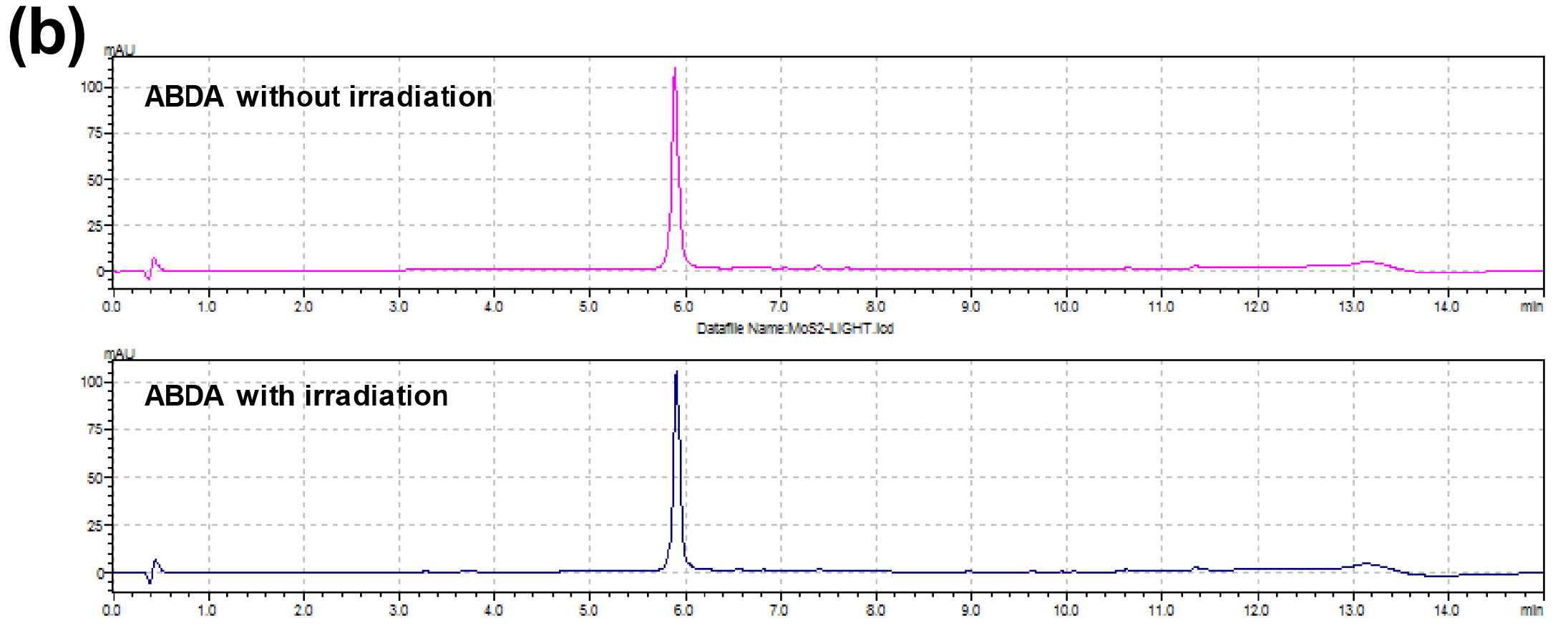


**Supplementary Figure 15.** ^1^H-NMR (a) and HPLC (b) spectrums of ABDA before and after irradiation for 8 mins.


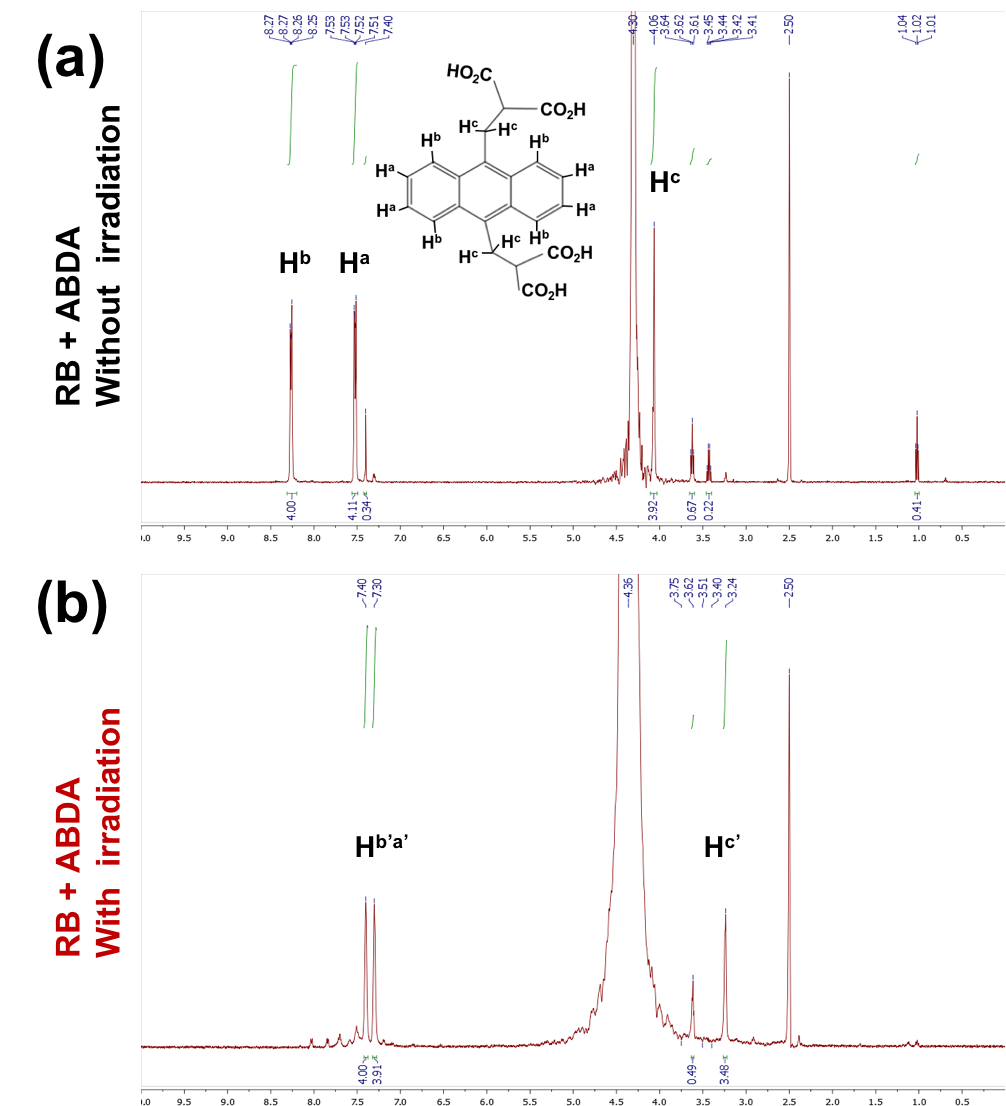


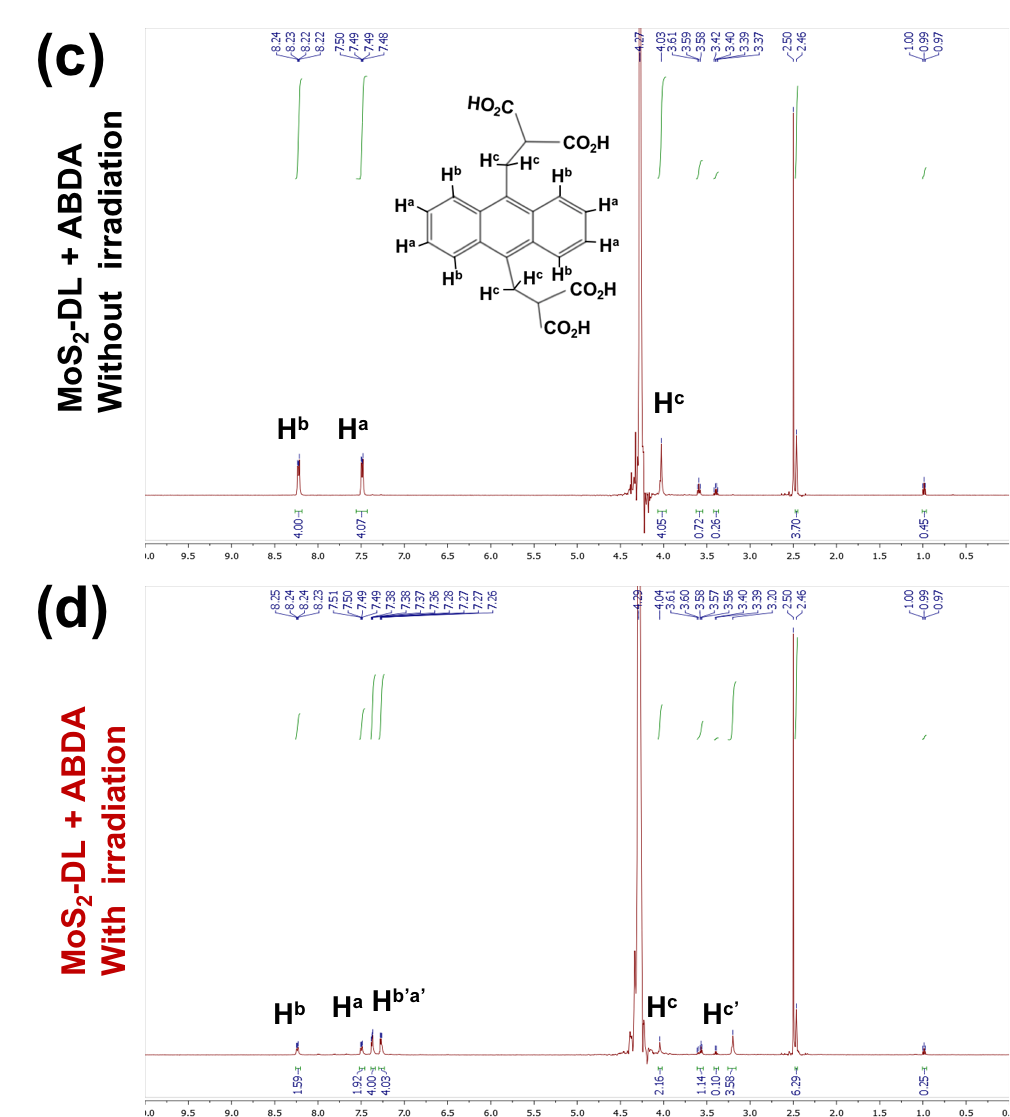


**Supplementary Figure 16**. 1H-NMR spectrum of the product of ABDA with and without light irradiation: (a) Incubated with Rose Bengal (RB) without irradiation, (b) Incubated with RB with irradiation, (c) Incubated with MoS_2_ QDs without irradiation and (d) Incubated with MoS_2_ QDs with irradiation. For the MoS_2_ QDs incubation group, the MoS_2_ QDs were removed by using centrifugal filters (MWCO 10K) before 1H-NMR testing.


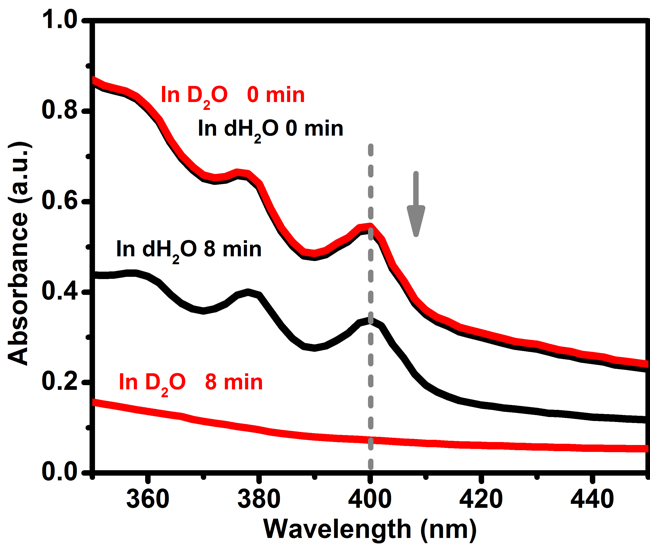


**Supplementary Figure 17**. Absorption spectrum of the mixture solution of MoS_2_-D_L_ QDs and ABDA in dH_2_O (black curve) and D_2_O (blue curve) before and after 8 min of light exposure.


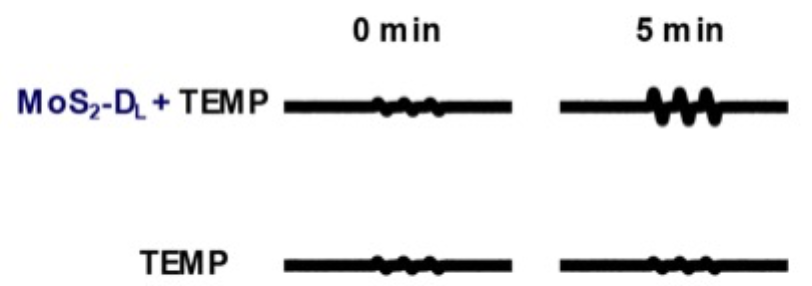


**Supplementary Figure 18.** ESR spectrum of MoS_2_ QDs in the presence of TEMP.


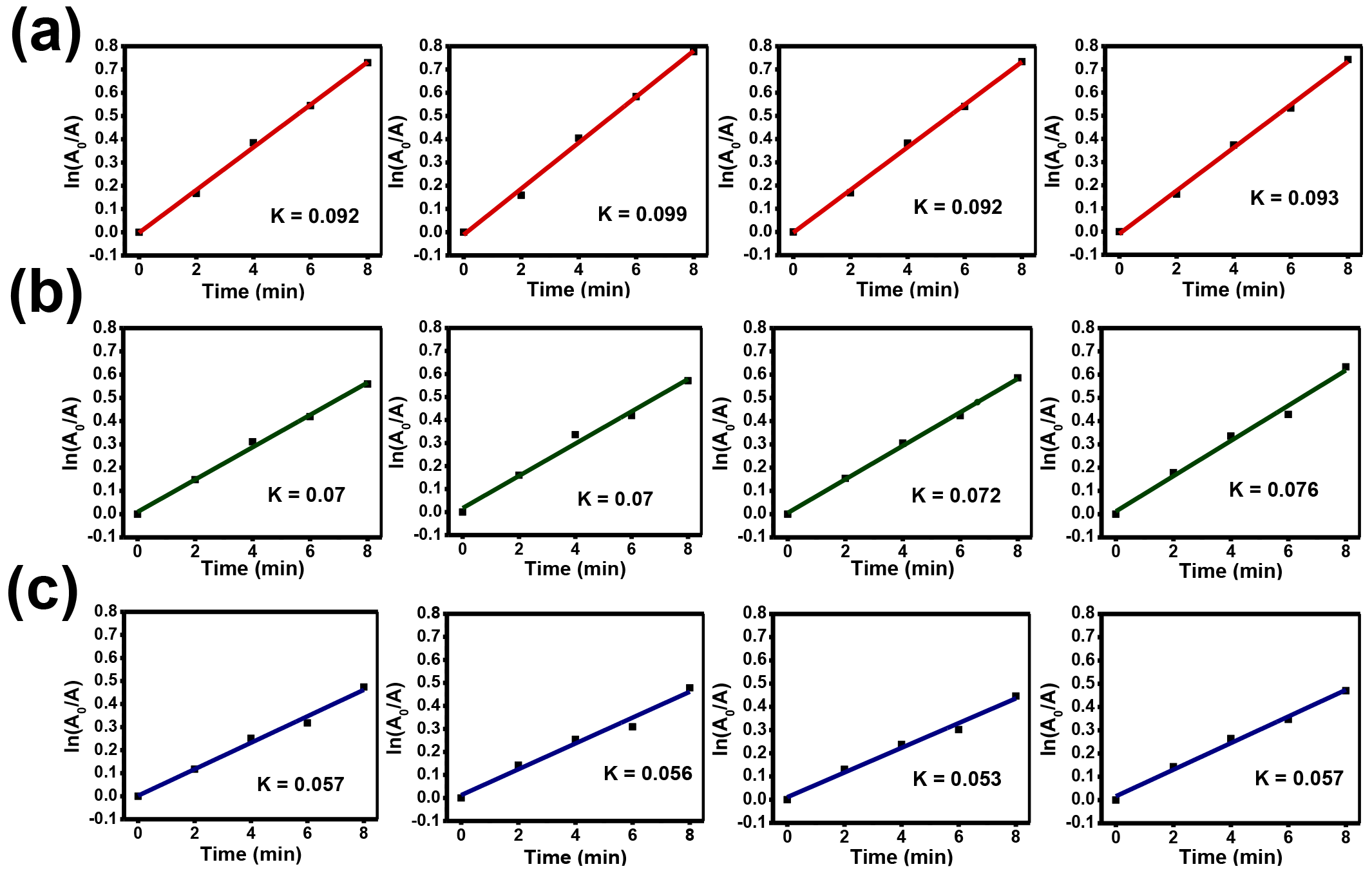


**Supplementary Figure 19**. Decomposition rate of the photosensitizing process of MoS_2_-D_H_ (a), MoS_2_-D_M_ (b) and MoS_2_-D_L_ (c) samples, where A_0_ is the absorbance of initial absorbance of ABDA and A is the absorbance of ABDA under light irradiation at different time points.


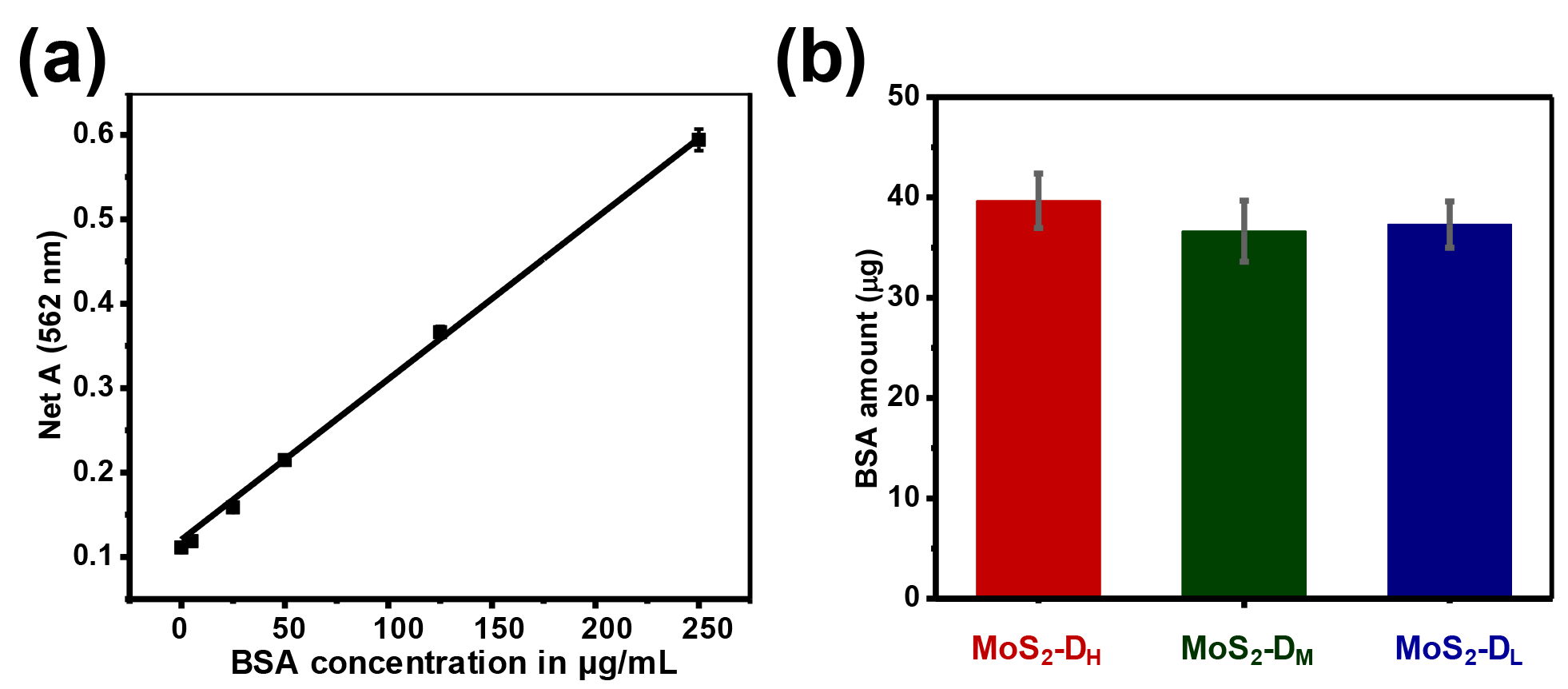


**Supplementary Figure 20.** (a) The quantification curve for BSA using the Bicinchoninic acid (BCA) reagent (37 ^o^C/30 min incubation);（b）There is insignificant difference between the BSA amounts on three MoS_2_ QDs defect types.


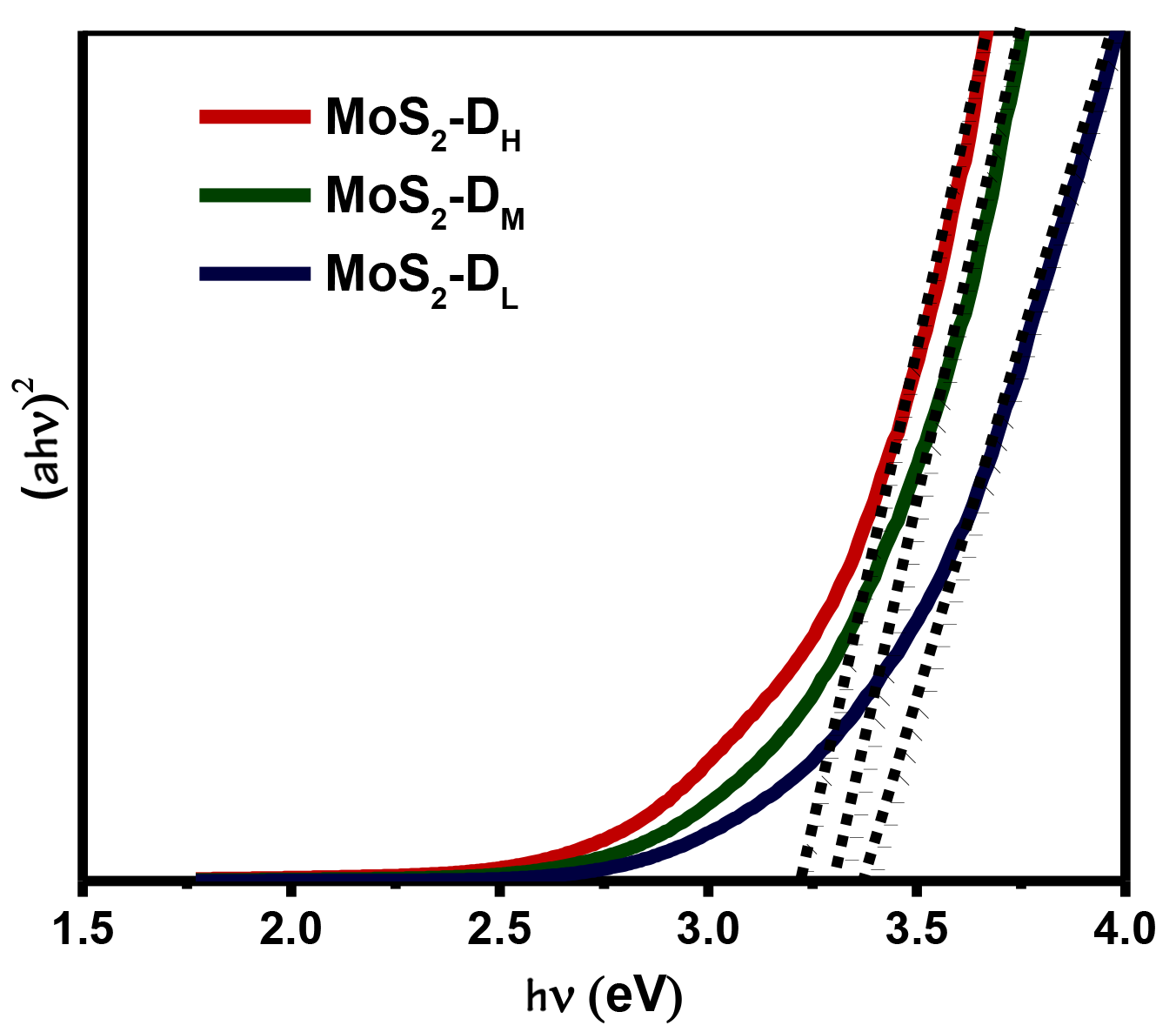


**Supplementary Figure 21.** Tauc plot αhν = A(hν-E_g_)^1/2^. Here α is the absorption coefficient, h is the Plank constant, ν is the photon frequency.


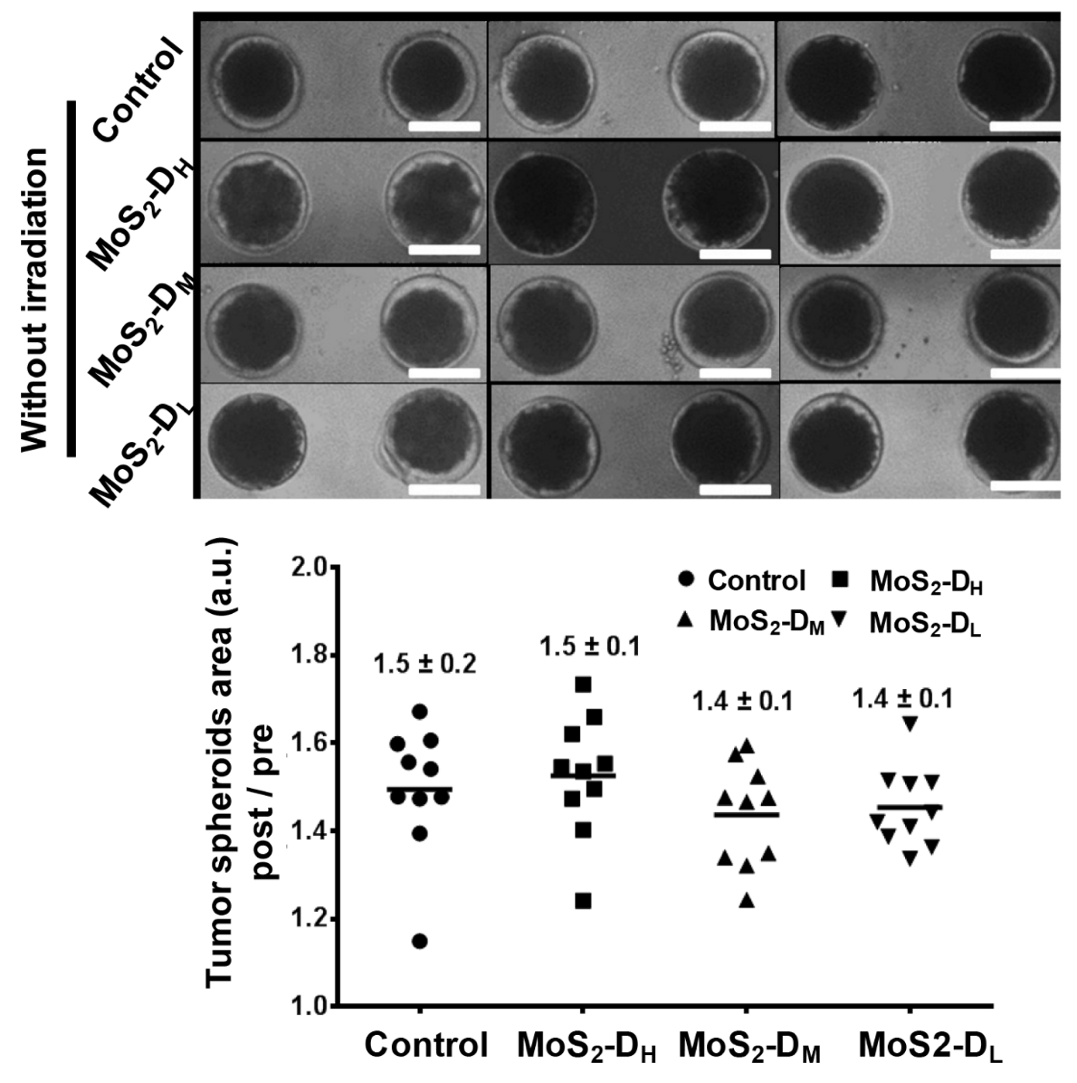


**Supplementary Figure 22.** 3D tumor spheroids were used to check the in vitro toxicity of defective MoS_2_ QDs without light irradiation. Mean ± SD, n=10, Scale bar: 100 μm.
