# Supplementary material for: Defect engineered bioactive transition metals dichalcogenides quantum dots
Source: Nat Commun. 2019 Jan 3;10:41. doi: 10.1038/s41467-018-07835-1 (PMC6318297; doi:10.1038/s41467-018-07835-1)
Supplement: Supplementary file 3 — Description of Additional Supplementary Files [file 41467_2018_7835_MOESM3_ESM.docx]

**Description of Additional Supplementary Files**

**File Name:** Supplementary Movie 1

**Description:** Real time video capture of synthesis steps of synthesizing MoS2 QDs. Aqueous reaction is fast, at room temperature and aqueous buffer.
